# Unification of the Nature’s Complexities via a Matrix Permanent—Critical Phenomena, Fractals, Quantum Computing, ♯P-Complexity

**DOI:** 10.3390/e22030322

**Published:** 2020-03-12

**Authors:** Vitaly Kocharovsky, Vladimir Kocharovsky, Sergey Tarasov

**Affiliations:** 1 Department of Physics and Astronomy, Texas A&M University, College Station, TX 77843-4242, USA; 2 Institute of Applied Physics, Russian Academy of Science, Nizhny Novgorod 603950, Russia; kochar@appl.sci-nnov.ru (V.K.); serge.tar@gmail.com (S.T.)

**Keywords:** ♯P-complexity, NP-complexity, critical phenomena, fractals, quantum computing, matrix permanent, MacMahon master theorem, Toeplitz determinant

## Abstract

We reveal the analytic relations between a matrix permanent and major nature’s complexities manifested in critical phenomena, fractal structures and chaos, quantum information processes in many-body physics, number-theoretic complexity in mathematics, and ♯P-complete problems in the theory of computational complexity. They follow from a reduction of the Ising model of critical phenomena to the permanent and four integral representations of the permanent based on (i) the fractal Weierstrass-like functions, (ii) polynomials of complex variables, (iii) Laplace integral, and (iv) MacMahon master theorem.

## 1. Introduction

We find a remarkable explicit connection between the major types of complexity in nature. They represent the critical phenomena, fractal structures in the theory of chaos, quantum information processing in many-body physics, cryptography, number-theoretic complexity in mathematics, and ♯P-complete problems in the theory of computational complexity. We show that all of them are analytically related to a well-known in mathematics matrix permanent via the fractal Weierstrass-like functions and polynomials or determinants involving complex variables.

The analysis is based on the concept of the ♯P/NP-complexity of computations and quantum information processing and computing (Section 2) as well as on a nontrivial reduction of the critical phenomena problem to a permanent (Section 3) and new integral representations of the permanent revealing its deep explicit relation to the fractals and chaos (Section 4), complex stochastic multivariate polynomials (Section 5), number-theoretical functions (Section 6), asymptotics of a Toeplitz determinant employed in the Onsager’s solution of Ising model and given by the Szegő limit theorems (Section 7).

The permanent of a n×n matrix $\left(A_{pq}\right)$ is defined similar to a determinant [1,2,3,4] via a sum running over permutations σ of 1,2,…,n, that is, over the symmetric group $S_{n}$,

(1) $$\mathrm{per}A=\sum_{\sigma \in S_{n}}\prod_{p=1}^{n}A_{p\sigma (p)}\;,\;\det A=\sum_{\sigma \in S_{n}}\mathrm{sgn}\left(\sigma \right)\prod_{p=1}^{n}A_{p\sigma (p)}.$$

## 2. The Matrix Permanent: Quantum Computing and ♯P-Complete Oracle

We start with remarks on the relation of the matrix permanent to the quantum many-body processes and computing.

### 2.1. The Permanent’s Complexity of the Quantum Information Processing and Computing

First of all, the permanent gives a result of the boson sampling in a multichannel quantum-optical network [5,6,7,8,9,10,11,12,13,14,15,16]—a simple prototype of a many-body quantum simulator. The latter amounts to evaluating the permanent of a unitary matrix of the channel couplings that could be addressed, for example, by an interesting method developed recently in Reference [11] on the basis of a complex phase-space representation. In particular, a possibility of a numerical computation of the permanent of the large-size (∼100×100) Fourier-transform matrices with applications in high-precision metrology was demonstrated in Reference [12].

The class of problems solvable in polynomial time by a quantum computer, BQP, is very wide and, likely, is not contained in the polynomial hierarchy PH [6]. (PH includes almost all complexity classes inside PSPACE (the problems solvable with a Polynomial SPACE) such as P, NP, co-NP, and even probabilistic classes such as BPP.) At the same time, computing of any quantum many-body process could be polynomial-time reducible to a matrix-permanent oracle. The latter is not proven yet. However, there is an explicit encoding of a transition amplitude of a quantum circuit in a universal quantum computer as the permanent of a matrix which is of size proportional to the number of quantum gates in the circuit [17]. An operator analog of the matrix permanent, a so-called quantum permanent, is directly related to characterizing an entanglement of a many-body system’s state which is considered as a main resource for quantum computing [18].

In cryptography, a permanent is capable for establishing a shared secret key via a public insecure channel [19].

### 2.2. The Permanent as the ♯P-Complete Oracle for the Universal Quantum Computing and the Toda’s Theorem

Computing the permanent is known to be one of the ♯P-complete problems [20,21] and, hence, would lead to a solution of every other ♯P- or NP-problem in polynomial time. On this basis, we suggest to employ the permanent as a convenient ♯P-complete oracle for the universal quantum computing if a specialized quantum simulator of the permanent would be realized. Of course, it requires finding a problem-specific algorithm of a deterministic polynomial-time Turing reduction to the matrix-permanent computing problem. Yet, this circumstance is common for any standard universal quantum computing of a particular problem that also requires finding a related, problem-specific algorithm. The choice of the permanent as the oracle is justified also by a surprising fact that the ♯P-problem of computing the permanent, contrary to many other ♯P-complete problems, corresponds to an easy, linear-time P problem on accepting paths [20].

The point is that the decision problem on an existence of a perfect matching for a given bipartite graph is soluble in polynomial time. Yet, the counting problem on the number of perfect matchings for the given bipartite graph is already ♯P-complete. The latter problem is known to be equivalent to the problem of computing the matrix permanent and was the first counting problem corresponding to an easy P problem shown to be ♯P-complete [20].

Computing the permanent involves a big fraction of hard instances, so that assuming its hardness on the worst case implies the problem’s hardness on average. The number of operations, ∼$n2^{n}$, required for computing the permanent by the best known deterministic algorithms of Ryser [22] or Glynn [23] grows exponentially with the matrix size *n*, contrary to only a polynomial number of operations ∼$n^{3}$ in a Gaussian elimination method for the matrix determinant. Even the world-fastest supercomputer Tianhe-2 cannot help to compute the permanent of a matrix with the size ∼60×60 or larger [8].

The permanent is deeply related to the concepts of the ♯P- and NP-complete problems in computational complexity theory [24] which are thought to require an exponentially long time of computation and, hence, cannot be solved by a classical computer. The ♯P- and NP-complete problems are those problems in the classes ♯P and NP, respectively, to each of which any other ♯P- or NP-problem can be reduced in polynomial time, and whose solution may still be verified in polynomial time. The complexity class ♯P is the class of counting problems on the number of accepting paths of a nondeterministic Turing machine running in polynomial time. It is different from a well-known NP-class of decision problems. We refer to the polynomial-time-relative-to-a-♯P-oracle class $P^{♯P}$ of problems soluble in polynomial time via an access to instantaneous answers to any counting problem in ♯P. The $P^{♯P}$ solves all quantum-computer problems [25], $BQP\subseteq P^{♯P}$.

The Toda’s theorem [26,27] states that the PH is contained in $P^{♯P}$, $PH\subseteq P^{♯P}$, and implies that for any problem in PH there is a deterministic polynomial-time Turing reduction to a counting problem. In other words, a polynomial-time machine with the ♯P-complete oracle can solve all problems in PH and, hence, all NP problems. In fact, the polynomial-time machine only needs to make one ♯P query to solve any problem in PH. This is an indication of the extreme difficulty of solving ♯P-complete problems exactly, for example, computing the permanent exactly.

Now we proceed with a reduction (2) of the Ising model to the permanent. A direct involvement of the Ising model and matrix permanent in the analysis of the many-body quantum-computing systems is important in view of a remarkable progress in the experimental realization of such systems, in particular, a programmable Ising-type quantum spin system with tunable interactions based on the reconfigurable arrays of up to 51 trapped cold atoms with strong interactions enabled by excitation to Rydberg states [28], a 100-spin Ising machine with all-to-all connections based on a network of optical parametric oscillators [29], and a proof of a photonic Ising machine [30], as well as due to Ising formulations of many NP problems [31].

## 3. Reduction of the Critical Phenomena to Computing a Matrix Permanent

Let us consider a generic model of the critical phenomena - the Ising model. We find the following analytic solution for the partition function and order parameter (a mean value of the *z*-component of a spin operator ${\hat{S}}_{r}$ at a site r),

(2) $$Z=\frac{\mathrm{per}A}{\det g_{1}},\;{\bar{S}}_{r}^{z}=\frac{\mathrm{per}{\left[A\right]}_{\left\{r\right\}}}{\mathrm{per}A}-\frac{1}{2},$$

for the Ising model of *N* spins $s=\frac{1}{2}$ located in a cubic lattice via a permanent of a circulant matrix $A=2+g_{1}^{-1}$. Correlation functions and other characteristics of the critical phenomena also can be expressed in a similar way. The result (2) is based on a bosonization of a many-body constrained system via a Holstein-Primakoff representation of spins. A matrix $g_{1rr^{\prime }}$ is a correlation function $g_{1}\left(r,r^{\prime }\right)$ of an auxiliary, much simpler system of the related unconstrained bosons and can be found by known methods. The submatrix ${\left[A\right]}_{\left\{r\right\}}$ differs from *A* by an absence of one row and one column which intersect at the entry $A_{rr}$.

This nontrivial constructive reduction of the Ising model to computing the permanent, that is a ♯P-problem, has been annotated in References [32,33]. It is derived in the present paper. In particular, it implies that a full analysis of the Ising model by numerical simulations alone is intractable. This fact stresses an importance of the exact general representation of the solution via a permanent, which unveils a remarkably canonical analytic structure of statistics and thermodynamics of the critical phenomena and guides to the adequate approximations and asymptotics for their computation including the known mean-field (random-phase) and renormalization-group approximations.

This novel approach can be compared with two other, more formal, plain approaches also pointing to a connection between the critical phenomena and permanent. One of them is a long known combinatorial method of obtaining all expansions and formulas of quantum field theory without diagrammatic expansions [34]. It stems from the basic representation of a many-particle wave function in a system of *N* Bose particles as a symmetrized product of the single-particle wave functions that is the Caianiello permanent. The latter is similar to the Slater determinant which represents a many-particle wave function in a system of *N* Fermi particles. The other approach is based on the graph theory. In particular, an ad hoc counting of all matchings of a bipartite graph representing a monomer-dimer model of phase transitions allows one to express its partition function via the permanent of a 0-1 matrix adjacent to the bipartite graph [35]. Importantly, the graph theory and the Markov chain Monte Carlo method provide a fully polynomial randomized approximation scheme (FPRAS) for numerical computation of the permanent of nonnegative matrices and a ferromagnetic Ising model [36,37,38,39]; for a discussion of a different scheme, see Reference [40].

For calculating the exact partition function, magnetization, Green’s functions (13), and other nonpolynomial averages, we employ a nonpolynomial diagram technique and partial operator contractions [32,41]. The point is that the constrained, true Green’s functions do not obey equations of a Dyson type due to a presence of the nonpolynomial functions $\theta (2s-{\hat{n}}_{r})$, and a standard diagram technique is not suited to deal with them.

### 3.1. The Constrained Spin Bosons in the Holstein-Primakoff Representation

Let us consider a 3d cubic lattice of *N* interacting quantized spins $s=\frac{1}{2}$ with a period *a* in a box with a volume $L^{3}$ and periodic boundary conditions. (The method is valid for an arbitrary dimensionality of the lattice d=1,2,3,…). The lattice sites are enumerated by a position vector r. According to the Holstein-Primakoff representation [42], worked out also by Schwinger [43], each spin is a system of two spin bosons, which are constrained to have a fixed total occupation

(3) $${\hat{n}}_{0r}+{\hat{n}}_{r}=2s;\;{\hat{n}}_{r}={\hat{a}}_{r}^{†}{\hat{a}}_{r},\;{\hat{n}}_{0r}={\hat{a}}_{0r}^{†}{\hat{a}}_{0r}.$$

The ${\hat{a}}_{r}$ and ${\hat{a}}_{0r}$ are the annihilation operators obeying the Bose canonical commutation relations: $\left[{\hat{a}}_{r},{\hat{a}}_{r^{\prime }}^{†}\right]=\delta_{r,r^{\prime }}$, $\left[{\hat{a}}_{0r},{\hat{a}}_{0r^{\prime }}^{†}\right]=\delta_{r,r^{\prime }}$, and all (r)-operators commute with all $\left(0r^{\prime }\right)$-operators; $\delta_{r,r^{\prime }}$ is a Kronecker delta. A vector spin operator ${\hat{S}}_{r}$ at a site r is given by its components:

(4) $${\hat{S}}_{r}^{x}=\frac{{\hat{a}}_{0r}^{†}{\hat{a}}_{r}+{\hat{a}}_{r}^{†}{\hat{a}}_{0r}}{2},\;{\hat{S}}_{r}^{y}=\frac{{\hat{a}}_{0r}^{†}{\hat{a}}_{r}-{\hat{a}}_{r}^{†}{\hat{a}}_{0r}}{2i},\;{\hat{S}}_{r}^{z}=s-{\hat{a}}_{r}^{†}{\hat{a}}_{r}.$$

A proper reduction of a many-body Hilbert space ensures [41] that this system is isomorphic to a system of *N* spin-boson excitations, described by annihilation operators ${\hat{\beta }}_{r}$ at each site r and obeying the Bose canonical commutation relations $\left[{\hat{\beta }}_{r},{\hat{\beta }}_{r^{\prime }}^{†}\right]=\delta_{r,r^{\prime }}$, if we cutoff them by a step-function $\theta (2s-{\hat{n}}_{r})$; θ(x)=1 if x≥0 and θ(x)=0 if x<0. This isomorphism is valid on an entire physically allowed Hilbert space and is achieved by equating the annihilation operators ${\hat{\beta }}_{r}^{{}^{\prime }}={\hat{\beta }}_{r}\theta \left(2s-{\hat{n}}_{r}\right)$ of those constrained, true excitations to the cutoff Holstein-Primakoff’s transition operators:

(5) $${\hat{\beta }}_{r}^{{}^{\prime }}={\hat{a}}_{0r}^{†}{\left(1+2s-{\hat{n}}_{r}\right)}^{-1/2}{\hat{a}}_{r}\theta \left(2s-{\hat{n}}_{r}\right).$$

Here and thereinafter we add a prime to a symbol of an unconstrained quantity to denote its cutoff, constrained counterpart. The vector components of the spin operator are

(6) $${\hat{S}}_{r}^{x}=\frac{1}{2}\left(S_{r}^{-}+{\hat{S}}_{r}^{+}\right),\;{\hat{S}}_{r}^{y}=\frac{i}{2}\left(S_{r}^{-}-{\hat{S}}_{r}^{+}\right),\;{\hat{S}}_{r}^{z}=s-{\hat{n}}_{r},$$

where the spin raising and lowering operators are equal to

(7) $${\hat{S}}_{r}^{+}=\sqrt{2s-{\hat{n}}_{r}}{\hat{\beta }}_{r}^{{}^{\prime }},\;{\hat{S}}_{r}^{-}={\hat{\beta }}_{r}^{{}^{\prime }†}\sqrt{2s-{\hat{n}}_{r}};\;{\hat{n}}_{r}={\hat{\beta }}_{r}^{{}^{\prime }†}{\hat{\beta }}_{r}^{{}^{\prime }}.$$

The aforementioned isomorphism is not trivial since it is not valid outside the constrained, physically allowed Hilbert space and the commutation relations for the creation and annihilation operators of the true spin excitations in Equations (5) are not canonical,

(8) $$\;\left[{\hat{\beta }}_{r}^{{}^{\prime }},{\hat{\beta }}_{r^{\prime }}^{{}^{\prime }†}\right]=\delta_{r,r^{\prime }}\left(1-\left(2s+1\right)\delta_{{\hat{n}}_{r},2s}\right).$$

A free Hamiltonian of a system of *N* spins in the lattice

(9) $$H_{0}=\sum_{r}\varepsilon {\hat{n}}_{r},\;{\hat{n}}_{r}={\hat{\beta }}_{r}^{†}{\hat{\beta }}_{r},\;\varepsilon =g\mu_{B}B_{ext},$$

is determined by a Zeeman energy $-g\mu_{B}B_{ext}{\hat{S}}^{z}$ of a spin in an external magnetic field $B_{ext}$ (which is assumed homogeneous and directed along the axis *z*) via a *g*-factor and a Bohr magneton $\mu_{B}=\frac{e\hbar }{2Mc}$. We intentionally define the free Hamiltonian in Equation (9) via the unconstrained occupation operators ${\hat{n}}_{r}={\hat{\beta }}_{r}^{†}{\hat{\beta }}_{r}$ on a full Fock space generated by a set of the creation operators $\left\{{\hat{\beta }}_{r}^{†}\right\}$, that is on the extended many-body Hilbert space without any $\theta (2s-{\hat{n}}_{r})$ cutoff factors. This makes the free Hamiltonian purely quadratic which is necessary for a validity of the standard diagram technique. The latter is crucial for a derivation of the Dyson-type equations, like Equation (15). One is allowed to skip the $\theta (2s-{\hat{n}}_{r})$ cutoff factors in $H_{0}$ in virtue of an equality ${\hat{\beta }}_{r}^{†}{\hat{\beta }}_{r}={\hat{\beta }}_{r}^{{}^{\prime }†}{\hat{\beta }}_{r}^{{}^{\prime }}$, valid on the physical many-body Hilbert space, and a fact that the occupation operator ${\hat{n}}_{r}={\hat{\beta }}_{r}^{†}{\hat{\beta }}_{r}$ leaves that space invariant.

An interaction Hamiltonian of the Ising model [44]

(10) $$H^{\prime }=-\sum_{r}\sum_{r^{\prime }\neq r}J_{r,r^{\prime }}{\hat{S}}_{r}^{z}{\hat{S}}_{r^{\prime }}^{z},$$

in view of the isomorphism’s Equations (5)–(7), takes a form

(11) $$H^{\prime }=-\sum_{r\neq r^{\prime }}J_{r,r^{\prime }}\left[s-\theta \left(2s-{\hat{n}}_{r}\right){\hat{n}}_{r}\right]\left[s-\theta \left(2s-{\hat{n}}_{r^{\prime }}\right){\hat{n}}_{r^{\prime }}\right].$$

Here a coupling between spins is a symmetric function $J_{r,r^{\prime }}=J_{r-r^{\prime }}$ of a vector $r-r^{\prime }$, connecting spins. For a spin at a site $r_{0}$ there are only the coordination number *p* of the nonzero couplings $J_{r_{0},r_{l}}\neq 0$ with the neighboring spins at sites $r_{l}=r_{0}+l;l=1,\ldots ,p$. The result in Equation (11) generalizes the Holstein-Primakoff’s one [42] by including the nonpolynomial operator $\theta (2s-{\hat{n}}_{r})$-cutoff functions, which add a spin-constraint nonlinear interaction and are crucially important in a critical region.

Since the Holstein-Primakoff’s paper of 1940, there were many unsuccessful attempts to convert it into a rigorous and tractable microscopic theory of critical phenomena in magnetic phase transitions. Note that a well-known Dyson’s theory of spin waves in a ferromagnet [45] is invalid in the critical region and restricts an analysis to just a well-formed ordered phase. Due to a lack of a proper mathematical apparatus, in particular, a technique of a partial contraction of operators and a diagram technique for the nonpolynomial averages, Dyson thought that “the Holstein-Primakoff formalism is thus essentially nonlinear and unamenable to exact calculations”.

A total Hamiltonian $H=H_{0}+H^{{}^{\prime }}$ defines, for any operator $\hat{A}$, a Matsubara operator ${\tilde{A}}_{\tau }=$$e^{\tau H}\hat{A}e^{-\tau H}$ evolving in an imaginary time $\tau \in [0,\frac{1}{T}]$ in a Heisenberg representation. A symbol *T* denotes a temperature. A symbol ${\tilde{A}}_{j\tau }$ stands for an operator itself ${\tilde{A}}_{1\tau }={\tilde{A}}_{\tau }$ at j=1 and a Matsubara-conjugated operator ${\tilde{A}}_{2\tau }={\tilde{\bar{A}}}_{\tau }$ at j=2.

The unconstrained and true Matsubara Green’s functions for spin excitations are defined by $T_{\tau }$-ordering [46]:

(12) $$G_{J_{1}}^{J_{2}}=-\langle T_{\tau }{\tilde{\beta }}_{J_{1}}{\tilde{\bar{\beta }}}_{J_{2}}\rangle ;\;J=\left\{j,\tau ,r\right\},$$

(13) $$G_{J_{1}}^{{}^{\prime }J_{2}}=-\langle T_{\tau }{\tilde{\beta^{\prime }}}_{J_{1}}{\tilde{\bar{\beta^{\prime }}}}_{J_{2}}\hat{\theta }\rangle /P_{s};\;P_{s}=\langle \hat{\theta }\rangle .$$

Here an unconstrained thermal average over an equilibrium statistical operator $\rho =e^{-\frac{H}{T}}/\mathrm{Tr}\left\{e^{-\frac{H}{T}}\right\}$ of the spin-boson excitations is denoted by the angles as

(14) $$\langle \cdots \rangle \equiv \mathrm{Tr}\left\{\cdots e^{-\frac{H}{T}}\right\}/\mathrm{Tr}\left\{e^{-\frac{H}{T}}\right\}$$

and a true, constrained thermal average is denoted as $\langle \cdots \hat{\theta }\rangle /P_{s}$. A partition function $P_{s}=\langle \hat{\theta }\rangle$ is equal to a cumulative probability of all occupations of the spin excitations in the unconstrained Fock space to be within physically allowed intervals $n_{r}\in \left[0,2s\right]$ for all lattice sites r; $\hat{\theta }=\prod_{r}\theta \left(2s-{\hat{n}}_{r}\right)$ is a product of all *N* cutoff factors.

In the Ising model there is no coherence, $\langle \beta_{r\tau }\rangle =0$, and the unconstrained Green’s functions obey the usual Dyson equation with a total irreducible self-energy $\Sigma_{j_{1}x_{1}}^{j_{2}x_{2}}$,

(15) $$\left(G_{j_{1}x_{1}}^{j_{2}x_{2}}\right)=\left(G_{j_{1}x_{1}}^{\left(0\right)j_{2}x_{2}}\right)+{\overset{ˇ}{G}}^{\left(0\right)}\left[\overset{ˇ}{\Sigma }\left[G_{j_{1}x_{1}}^{j_{2}x_{2}}\right]\right].$$

Here the integral operators $\overset{ˇ}{\Sigma }$ or ${\overset{ˇ}{G}}^{\left(0\right)}$, applied to any function $f_{jx}$ of an index *j* and a four-dimensional coordinate x={τ,r}, stand for a convolution of that function $f_{jx}$ over the variables j,τ,r with the total irreducible self-energy Σ or the free propagator $G^{\left(0\right)}$, respectively, for example,

(16) $$\overset{ˇ}{\Sigma }\left[G_{j_{1}x_{1}}^{j_{2}x_{2}}\right]\equiv \sum_{j=1}^{2}\sum_{r}\int_{0}^{1/T}\Sigma_{j_{1}x_{1}}^{jx}G_{jx}^{j_{2}x_{2}}d\tau .$$

The total irreducible self-energy is defined by an equation

(17) $$\langle T_{\tau }\left[{\tilde{\beta }}_{j_{1}x_{1}},{\tilde{H}}_{\tau_{1}}^{{}^{\prime }}\right]{\tilde{\bar{\beta }}}_{j_{2}x_{2}}\rangle ={\left(-1\right)}^{j_{1}}\sum_{j=1}^{2}\int_{0}^{\frac{1}{T}}\sum_{r}\Sigma_{j_{1}x_{1}}^{jx}G_{jx}^{j_{2}x_{2}}d\tau .$$

### 3.2. The Order Parameter and Correlation Functions via the True Probabilities of Spin-Boson Occupations

The magnetization at a lattice site r, being an order parameter of the Ising model, is equal to a true average of the spin *z*-component in Equation (6). For a spin $s=\frac{1}{2}$, it is

(18) $${\bar{S}}_{r}^{{}^{\prime }z}=1/2-\rho_{n_{r}=1}^{\prime },\;\rho_{n_{r}=n}^{\prime }=\langle \delta_{{\hat{n}}_{r},n}\hat{\theta }\rangle /P_{s},$$

that is determined by a $\hat{\theta }$-cutoff, true probability $\rho_{n_{r}=1}^{\prime }$ of a spin boson at site r to have one quantum of excitation.

The true, constrained correlation functions of the spin bosons, ${g^{\prime }}_{I_{1}}^{I_{2}}$, where I={j,r}, can be found as the Green’s functions $G_{J_{1}}^{{}^{\prime }J_{2}}$ in Equation (13) in the equal-time limit $\tau_{1}\to \tau_{2}-{\left(-1\right)}^{j_{2}}\times 0$. For a spin $s=\frac{1}{2}$, we find the exact solution for it,

(19) $${g^{\prime }}_{I_{1}}^{I_{2}}=-{\left(g^{-1}\right)}_{I_{1}}^{I_{2}}\left(1-\delta_{r_{1},r_{2}}\right)\rho_{n_{r_{1}}=0,n_{r_{2}}=0}^{\prime }-\rho_{n_{r_{1}}=0}^{\prime }\delta_{I_{1},I_{2}},$$

in terms of an unconstrained correlation matrix $g_{J}^{J^{\prime }}$ denoting an unconstrained Green’s function $G_{J}^{J^{\prime }}$ in Equation (12) for $\tau_{i}\neq \tau_{i^{\prime }}$ and its limit at $\tau_{i}\to \tau_{i^{\prime }}-{\left(-1\right)}^{j^{\prime }}\times 0$ for equal times in accord with the anti-normal ordering of operators ${\tilde{\beta }}_{J}$, ${\tilde{\bar{\beta }}}_{J^{\prime }}$. We consider a homogeneous phase, when the Green’s function $G_{j_{1}\tau_{1}r_{1}}^{j_{2}\tau_{2}r_{2}}$ depends on $r_{1}$ and $r_{2}$ only via $r_{2}-r_{1}$. It is a Toeplitz matrix with respect to indexes $r_{1}$, $r_{2}$. The matrix $g^{-1}$, which is inverse to the matrix $\left(g_{I_{1}}^{I_{2}}\right)$ of unconstrained correlations, can be calculated by a technique of Toeplitz matrices, known from the theory of the 2d Ising model [44,47,48,49,50,51,52]. In Equation (19), a quantity

(20) $$\rho_{n_{r_{1}}=n_{1},n_{r_{2}}=n_{2}}^{\prime }=\langle \delta_{{\hat{n}}_{r_{1}},n_{1}}\delta_{{\hat{n}}_{r_{2}},n_{2}}\hat{\theta }\rangle /P_{s}$$

stands for a true probability for two spin bosons at the sites $r_{1},r_{2}$ to have $n_{r_{1}}=n_{1}$ and $n_{r_{2}}=n_{2}$ quanta of excitations. Namely, Equation (19) involves a true probability for two spin-bosons to have zero quanta of excitations $n_{1}=n_{2}=0$ simultaneously.

### 3.3. The Unconstrained Probabilities of Spin-Boson Occupations via the Unconstrained Correlation Matrix

The next step is finding a joined non-cutoff probabilities of the spin-boson occupations at all *N* lattice sites

(21) $$\rho_{\left\{n_{r}\right\}}\equiv \langle \prod_{r=r_{1},\ldots ,r_{N}}\delta_{{\tilde{n}}_{r},n_{r}}\rangle .$$

Actually, for the reduction of the Ising model to a permanent we need to calculate just a particular joined probability

(22) $$\rho_{1\{m\}}\equiv \rho_{\left\{n_{r}=1:r=r_{1},\ldots ,r_{m};\;n_{r^{\prime }}=0:r^{\prime }\neq r_{1},\ldots ,r_{m}\right\}}=\langle f_{m}\rangle \;,$$

(23) $$f_{m}=\prod_{r=r_{k},k=1,\ldots ,m}\delta_{{\tilde{n}}_{r},1}\prod_{r^{\prime }\neq r_{k},k=1,\ldots ,m}\delta_{{\tilde{n}}_{r^{\prime }},0},$$

of getting unity occupations $n_{r_{k}}=1$ for *m* spin bosons at a subset of sites $\left\{m\right\}=\left\{r_{k},k=1,\ldots ,m\right\}$ and zero occupations for all other N−m spin bosons in the lattice, since the latter probability (22) determines the true joined statistics of the spin-boson occupations (calculated below in Section 3.4) and enters Equations (18) and (19) for the true magnetization and true correlation functions.

Also, for the self-energy in Equations (17) and (57), we need a similar joined unconstrained distribution of the spin-boson occupations at a subset of sites $\left\{M\right\}=\left\{r_{k},k=1,\ldots ,M\right\}$,

(24) $$\rho_{\left\{n_{r}\right\}}^{\left\{M\right\}}\equiv \langle \prod_{r=r_{1},\ldots ,r_{M}}\delta_{{\tilde{n}}_{r},n_{r}}\rangle ,\;M\leq N,$$

which admits arbitrary occupations $n_{r^{\prime }}=0,1,2,\ldots ,\infty$, $r^{\prime }\neq r_{1},\ldots ,r_{M},$ at all other N−M lattice sites, that is, it is non-cutoff averaged over the latter occupations. Again, we need just a particular joined probability

(25) $$\rho_{1\{m\}}^{\left\{M\right\}}\equiv \rho_{\left\{n_{r}=1:r=r_{1},\ldots ,r_{m};\;n_{r^{\prime }}=0:r^{\prime }=r_{m+1},\ldots ,r_{M}\right\}}^{\left\{M\right\}}=\langle f_{m}^{\left\{M\right\}}\rangle ,$$

(26) $$f_{m}^{\left\{M\right\}}=\prod_{r=r_{1},\ldots ,r_{m}}\delta_{{\tilde{n}}_{r},1}\prod_{r^{\prime }=r_{m+1},\ldots ,r_{M}}\delta_{{\tilde{n}}_{r^{\prime }},0},\;m\leq M\leq N,$$

of getting the unity occupations $n_{r_{k}}=1$ for *m* spin bosons at a subset of sites $\left\{m\right\}=\left\{r_{k},k=1,\ldots ,m\right\}\subseteq \left\{M\right\}$, zero occupations for M−m spin bosons at a subset of sites $\left\{M\right\}\setminus \left\{m\right\}=\left\{r_{k},k=m+1,\ldots ,M\right\}\subseteq \left\{M\right\}$, and arbitrary occupations for all other N−M spin bosons.

We employ the corresponding characteristic functions

(27) $$\Theta_{N}\left(\left\{u_{r}\right\}\right)=\langle \exp\left(i\sum_{r=r_{1},\ldots ,r_{N}}u_{r}{\tilde{n}}_{r}\right)\rangle ,$$

(28) $$\Theta_{N}^{\left\{M\right\}}\left(\left\{u_{r}\right\}\right)=\langle \exp\left(i\sum_{r=r_{1},\ldots ,r_{M}}u_{r}{\tilde{n}}_{r}\right)\rangle ,$$

the derivatives of which yield those joined probability distributions:

(29) $$\rho_{\left\{n_{r}\right\}}=\prod_{r=r_{1},\ldots ,r_{N}}\left(\frac{1}{n_{r}!}\frac{\partial^{n_{r}}}{\partial z_{r}^{n_{r}}}\right)\Theta_{N}{|}_{\left\{z_{r}=0\right\}},\;z_{r}=e^{iu_{r}},$$

(30) $$\rho_{\left\{n_{r}\right\}}^{\left\{M\right\}}=\prod_{r=r_{1},\ldots ,r_{M}}\left(\frac{1}{n_{r}!}\frac{\partial^{n_{r}}}{\partial z_{r}^{n_{r}}}\right)\Theta_{N}^{\left\{M\right\}}{|}_{\left\{z_{r}=0\right\}}.$$

We find the characteristic function $\Theta_{N}$ by means of the partial operator contraction within the nonpolynomial diagram technique [32,33] as follows

(31) $$\Theta_{N}\left(\left\{u_{r}\right\}\right)=\frac{1}{\sqrt{\det(g+Z_{\Theta })}}\prod_{r=r_{1},\ldots ,r_{N}}\frac{1}{1-z_{r}}=\frac{1}{\sqrt{\det g}}\frac{1}{\sqrt{\det(1-\left(1+g^{-1}\right)z)}},\;z_{I}^{I^{\prime }}=z_{r}\delta_{r,r^{\prime }}\delta_{j,j^{\prime }}.$$

Here the diagonal matrices $Z_{\Theta }$ and *z* are related as $Z_{\Theta }=z/\left(z-1\right)$. The obtained solution in Equation (31) is normalized to unity at a point $\left\{u_{r}=0\right\}$, $\Theta_{N}\left(\left\{u_{r}=0\right\}\right)=1$, as it should be for a characteristic function of any distribution.

The probability of the unity occupations for *m* spin bosons and zero occupations for all other spin bosons, Equation (22), is set by a differentiation of that characteristic function:

(32) $$\rho_{1\{m\}}=\frac{\partial^{m}\Theta_{N}}{\partial z_{r_{1}}\ldots \partial z_{r_{m}}}{|}_{\left\{z_{r}=0\right\}}.$$

It is a coefficient in front of the multilinear term $z_{r_{1}}\ldots z_{r_{m}}$ in a Taylor expansion of the characteristic function $\Theta_{N}$ over the variables $\left\{z_{r}\right\}$ at the zero point $\left\{z_{r}=0\right\}$.

In order to evaluate the Taylor expansion, we employ a well-known MacMahon master theorem [53,54]. It yields a Taylor expansion of a function, inversely proportional to a determinant of a matrix 1−Ax, over the variables $\left\{x_{i}\right\}$,

(33) $$\frac{1}{\det(1-Ax)}=\sum_{s_{1},\ldots ,s_{N}}{\mathrm{per}}^{\left(s_{1},\ldots ,s_{N}\right)}A\;x_{1}^{s_{1}}\ldots x_{N}^{s_{N}},$$

where $s_{i}\geq 0$ is a non-negative integer (i=1,…,N), $x=\mathrm{diag}\{x_{1},\ldots ,x_{N}\}$ a diagonal matrix, *A* a N×N matrix, and ${\mathrm{per}}^{\left(s_{1},\ldots ,s_{N}\right)}A$ a generalized permanent of the matrix *A*. For the required by Equation (32) multilinear terms with a subset of unity integers $\left\{s_{i}=1;i=1,\ldots ,m\right\}$ and the rest of integers being equal zero, the corresponding permanents are reduced to the standard permanent,

(34) $${\mathrm{per}}^{\left(\left\{s_{i}=1;i=1,\ldots ,m\right\},\left\{s_{j}=0;j=m+1,\ldots ,N\right\}\right)}A=\mathrm{per}A_{\left\{m\right\}},$$

of the corresponding (m×m)-submatrix $A_{\left\{m\right\}}$. In order to get the derivatives in Equation (32), one may compute a multilinear expansion of the characteristic function in Equation (31) by taking into account (i) a square-root function, additional to the MacMahon master Equation (33), via the corresponding Bell polynomials of that Fa$\overset{´}{a}$ di Bruno’s formula and (ii) an equality of the variables $z_{1r}^{1r}=z_{2r}^{2r}=z_{r}$ in the two adjacent columns with the same site-index r.

For simplicity’s sake, we consider below a case of vanishing anomalous correlations, $g_{1r}^{2r^{\prime }}=0$ for $\forall r,r^{\prime }$, and non-zero normal correlations $g_{1r}^{1r^{\prime }}=g_{2r}^{2r^{\prime }}$, which are real-valued in the homogeneous phases, when the matrix $g_{jr}^{j^{\prime }r^{\prime }}$ is a circulant Toeplitz matrix in indexes $r,r^{\prime }$ and the arbitrary phases of spin-bosons’ annihilation operators ${\hat{\beta }}_{r}$ are calibrated properly (for the calculations in a general case, see Reference [33]). In this case, one has $\det\left[g+Z_{\Theta }\right]={\left[\det\left[{\left(g+Z_{\Theta }\right)}_{1r}^{1r^{\prime }}\right]\right]}^{2}$ and Equations (31)–(34) yield

(35) $$\Theta_{N}=\frac{1}{\det[g_{1}-\left(1+g_{1}\right)z_{1}]}\;,\;g_{1}\equiv \left(g_{1r}^{1r^{\prime }}\right),\;z_{1}\equiv \left(z_{r}\delta_{r,r^{\prime }}\right),$$

$$\rho_{1\{m\}}=\frac{\mathrm{per}A_{\left\{m\right\}}^{\left(1\right)}}{\det g_{1}};\;A_{\left\{m\right\}}^{\left(1\right)}\equiv \left[{\left(1+g_{1}^{-1}\right)}_{r}^{r^{\prime }}\right];\;r,r^{\prime }=r_{1},\ldots ,r_{m}.$$

Here the elements of the (N×N)-matrices $g_{1}$ and $z_{1}$ as well as (m×m)-matrix ${\left(1+g_{1}^{-1}\right)}_{\left\{m\right\}}$ are labeled solely by the site-indexes $r,r^{\prime }$. The effect of the normal cross-correlations $g_{1r}^{1r^{\prime }}\neq 0$ between the spin bosons at different sites on their joined unconstrained statistics, described by Equation (35), remains highly nontrivial even in that case of vanishing anomalous correlations $g_{1r}^{2r^{\prime }}=0$ for $\forall r,r^{\prime }$.

For the joined unconstrained, non-cutoff distribution of the spin-boson occupations at only a subset of lattice sites $\left\{M\right\}=\left\{r_{k},k=1,\ldots ,M\right\},M\leq N$, defined in Equation (24), a derivation is similar. We just need to restrict the (2N×2N)-matrices $g,Z_{\Theta }$, *z* to the corresponding quasi-diagonal (2M×2M)-block matrices $g_{\left\{M\right\}},Z_{\Theta }^{\left\{M\right\}}$, $z_{\left\{M\right\}}$;

(36) $$Z_{\Theta }^{\left\{M\right\}}=\mathrm{diag}\left\{{\left(Z_{\Theta }\right)}_{I}^{I};r=r_{1},\ldots ,r_{M};j=1,2\right\}\equiv \frac{z_{\left\{M\right\}}}{z_{\left\{M\right\}}-1}.$$

The result for the characteristic function

(37) $$\Theta_{N}^{\left\{M\right\}}\left(\left\{u_{r}\right\}\right)=\frac{1}{\sqrt{\det(g_{\left\{M\right\}}+Z_{\Theta }^{\left\{M\right\}})}}\prod_{r=r_{1}}^{r_{M}}\frac{1}{1-z_{r}}=\frac{1}{\sqrt{\det g_{\left\{M\right\}}}}\frac{1}{\sqrt{\det(1-\left(1+g_{\left\{M\right\}}^{-1}\right)z_{\left\{M\right\}})}}$$

is similar to Equation (31). Obviously, its differentiation,

(38) $$\rho_{1\{m\}}^{\left\{M\right\}}=\frac{\partial^{m}\Theta_{N}^{\left\{M\right\}}}{\partial z_{r_{1}}\ldots \partial z_{r_{m}}}{|}_{\left\{z_{r}=0\right\}},$$

yields the corresponding, similar to Equation (32), unconstrained probabilities for the spin bosons to have the unity occupations at the subset of lattice sites $\left\{m\right\}=\{r_{i},i=1,\ldots ,$m},m≤M≤N, zero occupations at the subset of lattice sites $\left\{M\right\}\setminus \left\{m\right\}=\left\{r_{k},k=m+1,\ldots ,M\right\}$, and arbitrary occupations, $n_{r^{\prime }}=0,1,2,\ldots ,\infty$, at the rest N−M sites.

The derived characteristic functions in Equations (31) and (37) immediately yield the probability for all *N* or for a subset $\left\{M\right\}=\left\{r_{k},k=1,\ldots ,M\right\},M\leq N$, of the spin bosons to have zero occupations:

(39) $$\rho_{0}^{\left\{N\right\}}\equiv \langle f_{0}\rangle =\frac{1}{\sqrt{\det g}},\;\rho_{0}^{\left\{M\right\}}\equiv \langle f_{0}^{\left\{M\right\}}\rangle =\frac{1}{\sqrt{\det g_{\left\{M\right\}}}}.$$

Finally, we present the explicit formulas for the characteristic functions of the joined non-cutoff probability distributions in the most important cases of the single-site (M=1) and two-sites (M=2) subsets of the spin bosons:

(40) $$\Theta_{N}^{\left\{1\right\}}\left(u_{r}\right)=\frac{\rho_{0}}{\sqrt{\det(1-\left(1+S^{-1}\right)z_{\left\{1\right\}})}},$$

(41) $$\Theta_{N}^{\left\{2\right\}}\left(u_{r_{1}},u_{r_{2}}\right)=\frac{\rho_{0,0}}{\sqrt{\det(1-\left(1+q^{-1}\right)z_{\left\{2\right\}})}}.$$

### 3.4. The Partition Function and the True Probabilities of Spin-Boson Occupations

Here we give the exact analytic formulas for the partition function and true joined probability distributions $\rho_{n_{r}}^{\prime }$, $\rho_{n_{r_{1}},n_{r_{2}}}^{\prime }$, $\rho_{\left\{n_{r}\right\}}^{\prime }$ of the physically allowable spin-boson occupations $n_{r}=0,1$. Those distributions are simply the $\hat{\theta }$-cutoff versions of the unconstrained distributions $\rho_{n_{r}}$, $\rho_{n_{r_{1}},n_{r_{2}}}$, $\rho_{\left\{n_{r}\right\}}$, restricted to the unity-occupation ones $\rho_{1\{m\}}$ in Equation (32). Note that the unconstrained occupation distributions, calculated in the Section 3.3, already contain all effects of the constraints and spin interaction, except the $\hat{\theta }$-cutoff only, since they were calculated for the exact, constrained and $\hat{\theta }$-cutoff, Hamiltonian (11).

We start the analysis of the true joined distribution of the occupations $\left\{n_{r}=0\;\mathrm{or}\;1\right\}$ for all *N* spin bosons,

(42) $$\rho_{\left\{n_{r}\right\}}^{\prime }\equiv \frac{1}{P_{s}}\langle \prod_{r}\delta_{{\hat{n}}_{r},n_{r}}\hat{\theta }\rangle ,\;P_{s}=\langle \hat{\theta }\rangle ,$$

with an evaluation of the partition function $P_{s}$ from Equation (13). It is equal to the sum of the probabilities $\rho_{1\{m\}}$ in Equation (32) over all occupation configurations $\left\{n_{r}=0\;\mathrm{or}\;1;\;r=r_{1},\ldots ,r_{N}\right\}$, which can be written as follows

(43) $$P_{s}=\frac{\partial^{N}}{\partial z_{r_{1}}\ldots \partial z_{r_{N}}}\left[\Theta_{N}\prod_{r=r_{1},\ldots ,r_{N}}\left(1+z_{r}\right)\right]{|}_{\left\{z_{r}=0\right\}}=\frac{\partial^{N}\Theta^{\prime }}{\partial z_{r_{1}}\ldots \partial z_{r_{N}}}{|}_{\left\{z_{r}=0\right\}},\;\Theta^{\prime }=\frac{1}{\sqrt{\det g}}\frac{1}{\sqrt{\det[1-\left(2+g^{-1}\right)z]}}.$$

The second equality in the equation for $P_{s}$ is due to the fact that the terms with the square, $z_{r}^{2}$, and higher powers of any variable $z_{r}$ do not contribute to the considered derivative at the zero point $\left\{z_{r}=0\right\}$. Note that the newly introduced function $\Theta^{\prime }$ differs from the characteristic function $\Theta_{N}$ in Equation (31) only by a substitution of the matrix $A=1+g^{-1}$ with the matrix

(44) $$A^{\prime \prime }=2+g^{-1}.$$

Thus, an evaluation of the partition function $P_{s}$ can be done similar to the evaluation of the probability $\rho_{1\{m\}}$ at m=N described above. In particular, in the case of the vanishing anomalous correlations $g_{1r}^{2r^{\prime }}=0$ and non-zero normal correlations $g_{1r}^{1r^{\prime }}=g_{2r}^{2r^{\prime }}$, as in Equation (35), we find

(45) $$P_{s}=\frac{\mathrm{per}(2+g_{1}^{-1})}{\det g_{1}},\;g_{1}\equiv g_{1\{N\}}\equiv \left[g_{1r}^{1r^{\prime }}\right].$$

A result for the single-site zero occupation probability

(46) $$\rho_{n_{r_{1}}=0}^{\prime }=\frac{1}{P_{s}}\frac{\partial^{N-1}\Theta^{\prime }}{\partial z_{r_{2}}\ldots \partial z_{r_{N}}}{|}_{\left\{z_{r}=0\right\}}$$

differs from $P_{s}$ only by an absence of one partial derivative $\partial /\partial z_{r_{1}}$ and by a normalization factor. In particular, in the case of the vanishing anomalous correlations $g_{1r}^{2r^{\prime }}=0$ and non-zero normal correlations $g_{1r}^{1r^{\prime }}=g_{2r}^{2r^{\prime }}$, we have

(47) $$\rho_{n_{r_{1}}=0}^{\prime }=\frac{\mathrm{per}{\left(2+g_{1}^{-1}\right)}_{\left\{N-1\right\}}}{\mathrm{per}(2+g_{1}^{-1})};\;{\left(2+g_{1}^{-1}\right)}_{\left\{N-1\right\}}\equiv \left({\left(2+g_{1}^{-1}\right)}_{r}^{r^{\prime }}\right),\;r,r^{\prime }\neq r_{1}.$$

The true single-site unity occupation probability is equal

(48) $$\rho_{n_{r_{1}}=1}^{\prime }=1-\rho_{n_{r_{1}}=0}^{\prime }.$$

The true two-sites zero occupation probability

(49) $$\rho_{n_{r_{1}}=0,n_{r_{2}}=0}^{\prime }=\frac{1}{P_{s}}\frac{\partial^{N-2}\Theta^{\prime }}{\partial z_{r_{3}}\ldots \partial z_{r_{N}}}{|}_{\left\{z_{r}=0\right\}}$$

differs from the single-site one in Equation (46) only by an absence of one more partial derivative $\partial /\partial z_{r_{2}}$. So, in the case of the vanishing anomalous correlations and non-zero normal correlations $g_{1r}^{1r^{\prime }}=g_{2r}^{2r^{\prime }}$, as in Equation (35), one has

(50) $$\rho_{n_{r_{1}}=0,n_{r_{2}}=0}^{\prime }=\frac{\mathrm{per}{\left(2+g_{1}^{-1}\right)}_{\left\{N-2\right\}}}{\mathrm{per}(2+g_{1}^{-1})};\;{\left(2+g_{1}^{-1}\right)}_{\left\{N-2\right\}}\equiv \left({\left(2+g_{1}^{-1}\right)}_{r}^{r^{\prime }}\right),\;r,r^{\prime }\in \left\{r_{3},\ldots ,r_{N}\right\}.$$

The true two-sites probabilities for other occupation combinations can be computed from the probabilities, presented above, as follows

(51) $$\begin{array}{c} \rho_{n_{r_{1}}=0,n_{r_{2}}=1}^{\prime }=\rho_{n_{r_{1}}=0}^{\prime }-\rho_{n_{r_{1}}=0,n_{r_{2}}=0}^{\prime }\;,\\ \rho_{n_{r_{1}}=1,n_{r_{2}}=1}^{\prime }=\rho_{n_{r_{1}}=1}^{\prime }-\rho_{n_{r_{1}}=1,n_{r_{2}}=0}^{\prime }\;. \end{array}$$

These equations stem from a fact that the true single-site occupation distribution is equal to the true two-sites occupation distribution, averaged over the physically allowable occupations $n_{r_{2}}=0,1$ of a spin boson at the second site:

(52) $$\rho_{n_{r_{1}}}^{\prime }=\rho_{n_{r_{1}},n_{r_{2}}=0}^{\prime }+\rho_{n_{r_{1}},n_{r_{2}}=1}^{\prime }.$$

The true three-sites and other multiple-sides occupation distributions are not required for calculating the true order parameter and correlation functions, but are necessary for the analysis of the true multiple-sides correlations and statistics. Those m-sides occupation distributions can be computed in a similar way by an induction:

(53) $$\rho_{n_{r_{1}}=0,\ldots ,n_{r_{m}}=0}^{\prime }=\frac{1}{P_{s}}\frac{\partial^{N-m}\Theta^{\prime }}{\partial z_{r_{m+1}}\ldots \partial z_{r_{N}}}{|}_{\left\{z_{r}=0\right\}},\;m\leq N,$$

(54) $$\rho_{n_{r_{1}},\ldots ,n_{r_{m-1}},n_{r_{m}}=1}^{\prime }=\rho_{n_{r_{1}},\ldots ,n_{r_{m-1}}}^{\prime }-\rho_{n_{r_{1}},\ldots ,n_{r_{m-1}},n_{r_{m}}=0}^{\prime }.$$

In the case of the vanishing anomalous correlations $g_{1r}^{2r^{\prime }}=0$ and non-zero normal correlations $g_{1r}^{1r^{\prime }}=g_{2r}^{2r^{\prime }}$, one has

(55) $$\rho_{n_{r_{1}}=0,\ldots ,n_{r_{m}}=0}^{\prime }=\frac{\mathrm{per}{\left(2+g_{1}^{-1}\right)}_{\left\{N-m\right\}}}{\mathrm{per}(2+g_{1}^{-1})};\;{\left(2+g_{1}^{-1}\right)}_{\left\{N-m\right\}}\equiv \left({\left(2+g_{1}^{-1}\right)}_{r}^{r^{\prime }}\right),\;r,r^{\prime }\in \left\{r_{m+1},\ldots ,r_{N}\right\}.$$

We stress, that the true joined distributions of the spin-boson occupations, even for a subset of lattice sites $\left\{M\right\}=\left\{r_{k},k=1,\ldots ,M\right\},M\leq N$, always are determined by the full (2N×2N)-matrix $g^{-1}$, which is inverse to the 2N×2N equal-time anti-normally ordered correlation matrix *g*. This is in contrast with the unconstrained joined distributions in Equations (37) and (38), which are determined only by the corresponding quasi-diagonal (2M×2M)-block $g_{\left\{M\right\}}$ of the full (2N×2N)-matrix *g*.

A detailed analysis of the true spin-boson occupation probability distributions obtained above as well as the true order parameter and correlation functions in Equations (18) and (19) will be given elsewhere, since they are not required for finding the self-energy in Section 3.5 and the exact self-consistency equation in Section 3.6 below.

### 3.5. The Exact Solution for the Total Irreducible Self-Energy via the Unconstrained Correlation Matrix

In order to find the aforementioned spin-boson occupation probabilities and the correlation matrix $g_{J}^{J^{\prime }}$, it is crucial to get an exact solution to Equation (17) for the total irreducible self-energy which allows one to go beyond standard second-order or ladder approximations. For a given site $r_{0}$ in a lattice and any its nearest-neighbor site $r_{l}$, l=1,…,p, we introduce a correlation (4×4)-matrix

(56) $$q_{I}^{I^{\prime }}\left(l\right)\equiv g_{jR}^{j^{\prime }R^{\prime }}=-\langle A{\hat{\beta }}_{jR}{\hat{\beta }}_{j^{\prime }R^{\prime }}^{†}\rangle ,\;q\left(l\right)=\left(\frac{S\;\;|\;C}{C^{†}|\;S}\right).$$

Here $q=q^{†}$ is hermitian, R and $R^{\prime }$ run over two values $\left\{r_{0},r_{l}\right\}$, A means anti-normal ordering, (2×2)-matrices $g_{j}^{j^{\prime }}\left(l\right)=g_{jr_{0}}^{j^{\prime }r_{l}}$ of basis auto- and cross-correlations are denoted as $g\left(0\right)=S=S^{†}$ and g(l≠0)=C(l), respectively. An exact solution for the self-energy is a matrix 2(p+1)-banded in indexes $I_{0}=\left\{j_{0},r_{0}\right\}$ and I={j,r},

(57) $$\Sigma_{J_{0}}^{J}=\delta \left(\tau -\tau_{0}\right)\sum_{l=0}^{p}\delta_{r,r_{l}}\Sigma_{j_{0}r_{0}}^{jr_{l}}\left(l\right),\;r_{l}=r_{0}+l,$$

where the (2×2)-matrix blocks $\Sigma \left(l\right)=(\Sigma_{j_{0}r_{0}}^{jr_{l}}\left(l\right))$ are

(58) $$\begin{array}{c} \Sigma \left(0\right)=\sum_{l=1}^{p}J_{r_{0},r_{l}}[\rho_{1}S^{-1}+\rho_{0}S^{-2}-2\rho_{1,1}K-2\rho_{0,1}K^{2}\\ -2\rho_{1,0}KCS^{-2}C^{†}K+2\rho_{0,0}K\left(KCS^{-2}C^{†}+CS^{-2}C^{†}K\right)K], \end{array}$$

(59) $$\Sigma \left(l\neq 0\right)=2J_{r_{0},r_{l}}\left[\left(\rho_{1,1}+\rho_{0,1}K+\rho_{0,0}KC\frac{1}{S^{2}}C^{†}K\right)KC\frac{1}{S}+\left(\rho_{1,0}-\rho_{0,0}K\right)KC\frac{1}{S^{2}}\left(1+C^{†}KC\frac{1}{S}\right)\right];$$

$$K=\frac{1}{S-CS^{-1}C^{†}}.$$

Here $\rho_{n_{r_{0}}}=\langle \delta_{{\tilde{n}}_{r_{0}},n_{r_{0}}}\rangle$ and $\rho_{n_{r_{0}},n_{r_{l}}}=\langle \delta_{{\tilde{n}}_{r_{0}},n_{r_{0}}}\delta_{{\tilde{n}}_{r_{l}},n_{r_{l}}}\rangle$ are the non-cutoff probabilities for the spin bosons at the sites $r_{0}$ and $r_{l}$ to acquire $n_{r_{0}}$ and $n_{r_{l}}$ quanta of excitations. The probabilities of the zero occupations follow from Equation (39):

(60) $$\rho_{0}\equiv \rho_{0}^{\left\{1\right\}}=1/\sqrt{\det S},\;\rho_{0,0}\equiv \rho_{0}^{\left\{2\right\}}=1/\sqrt{\det q}.$$

For the single-site probability one has M=1 and (2×2)-matrix $g_{\left\{1\right\}}=S$, Equation (56), so that Equations (38) and (40) yield

(61) $$\rho_{1}\equiv \rho_{1\{1\}}^{\left\{1\right\}}=\rho_{0}\left[1+{\left(S^{-1}\right)}_{1r}^{1r}\right]\equiv \rho_{0}\left[1+\frac{g_{1r}^{1r}}{\det S}\right].$$

For the two-sites probabilities one has M=2 and (4×4)-matrix $g_{\left\{2\right\}}=q$ (where hereinafter a matrix $q_{jR}^{j^{\prime }R^{\prime }}$ is defined similar to Equation (56) with the R and $R^{\prime }$ running over arbitrary two sites $\left\{r_{1},r_{2}\right\}$, not necessarily neighboring sites), and Equations (38) and (41) yield

(62) $$\rho_{1,0}\equiv \rho_{1\{1\}}^{\left\{2\right\}}=\rho_{0,0}\left[1+{\left(q^{-1}\right)}_{1r_{1}}^{1r_{1}}\right]\equiv \rho_{0,0}\left[1+\frac{\det{\left[q\right]}_{1r_{1}}^{1r_{1}}}{\det q}\right],$$

(63) $$\rho_{0,1}\equiv \rho_{1\{1\}}^{\left\{2\right\}}=\rho_{0,0}\left[1+{\left(q^{-1}\right)}_{1r_{2}}^{1r_{2}}\right]\equiv \rho_{0,0}\left[1+\frac{\det{\left[q\right]}_{1r_{2}}^{1r_{2}}}{\det q}\right],$$

(64) $$\rho_{1,1}\equiv \rho_{1\{2\}}^{\left\{2\right\}}=\rho_{0,0}\left\{\left[1+{\left(q^{-1}\right)}_{1r_{1}}^{1r_{1}}\right]\left[1+{\left(q^{-1}\right)}_{1r_{2}}^{1r_{2}}\right]+{\left(q^{-1}\right)}_{1r_{1}}^{1r_{2}}{\left(q^{-1}\right)}_{1r_{2}}^{1r_{1}}+{\left(q^{-1}\right)}_{1r_{1}}^{2r_{2}}{\left(q^{-1}\right)}_{2r_{2}}^{1r_{1}}\right\},$$

where ${\left[q\right]}_{I}^{I^{\prime }}$ stands for a $II^{\prime }$-submatrix of *q*, that is, for the matrix *q* with the *I*-th row and $I^{\prime }$-th column deleted.

### 3.6. The Exact Closed Self-Consistency Equation for the Unconstrained Correlation Matrix

Now we can make a final, crucial step in the exact reduction of the Ising model to the matrix permanent—find an exact closed self-consistency equation for the nearest-neighbors’, basis normal and anomalous auto- and cross-correlations $g_{1r_{0}}^{1r_{l}}=g_{2r_{0}}^{2r_{l}*},\;g_{1r_{0}}^{2r_{l}}=g_{2r_{0}}^{1r_{l}*},l=0,1,\ldots ,p$, in Equation (56). Indeed, the total irreducible self-energy in Equation (57) and the spin-boson unconstrained occupation probabilities, Equations (60)–(64), entering formulas for the self-energy, are known exactly via the (1+p) basis correlation (2×2)-matrices g(l),l=0,1,…,p, Equation (56), that is, the matrix $S\equiv g\left(0\right)=\left(g_{jr_{0}}^{j^{\prime }r_{0}}\right)$ of the auto-correlations for a spin boson at the site $r_{0}$ and the coordination number *p* matrices $C\left(l\right)\equiv g\left(l\neq 0\right)=\left(g_{jr_{0}}^{j^{\prime }r_{l}}\right)$ of the cross-correlations of a spin boson at the site $r_{0}$ with the nearest-neighbors at the sites $r_{l}=r_{0}+l$. Due to the complex-conjugation relations

(65) $$g_{1r_{0}}^{1r_{0}}=g_{2r_{0}}^{2r_{0}},\;g_{1r_{0}}^{2r_{0}}=g_{2r_{0}}^{1r_{0}*},\;g_{1r_{0}}^{1r_{l}}=g_{2r_{0}}^{2r_{l}*},\;g_{1r_{0}}^{2r_{l}}=g_{2r_{0}}^{1r_{l}*},$$

there are only two independent, normal $g_{1r_{0}}^{1r_{l}}$ and anomalous $g_{1r_{0}}^{2r_{l}}$, correlation parameters per each basis correlation (2×2)-matrix, that is, only 2(1+p) numbers, which determine all details of the critical phenomena.

Thus, we can find the self-consistency equation for those 2(1+p) basis auto- and cross-correlations in two steps. First, we solve the Dyson-type Equation (15) for the unconstrained Green’s functions in terms of those basis correlations. Second, we close the loop by expressing the basis correlations themselves via those Green’s functions.

For the considered stationary homogeneous phases, the Green’s functions, the equal-time correlation functions, and the self-energy depend only on the differences of their arguments $\tau =\tau_{1}-\tau_{2}$ and $r=r_{2}-r_{1}$, that is,

(66) $$G_{J_{1}}^{J_{2}}=G_{j_{1}j_{2}}\left(\tau ,r\right),\;g_{j_{1}r_{1}}^{j_{2}r_{2}}=g_{j_{1}j_{2}}\left(r\right),\;\Sigma_{J_{1}}^{J_{2}}=\delta \left(\tau \right)\Sigma_{j_{1}j_{2}}\left(r\right).$$

Hence, it is straightforward to solve the Dyson-type Equation (15) by means of the Fourier transformation over the imaginary time $\tau \in [-\frac{1}{T},\frac{1}{T}]$ and the discrete Fourier transformation over the space. The latter has a following form

(67) $$g\left(k\right)=\sum_{r}g\left(r\right)e^{-ikr},\;g\left(r\right)={\left(\frac{a}{L}\right)}^{d}\sum_{k}g\left(k\right)e^{ikr},$$

where the sums run over all lattice sites r with a period *a* and discrete wave vectors $k=\left\{k_{i}|i=1,\cdots ,d\right\},k_{i}=\frac{2\pi }{L}q$ with an integer *q*; $k_{i}\in \left[-\frac{\pi }{a},\frac{\pi }{a}\right]$. We discern the Fourier transform and its inverse by the arguments k and r. A result for the normal and anomalous Green’s functions is

(68) $$G_{11}\left(\tau ,k\right)=\sum_{j=1}^{2}{\left(-1\right)}^{j}\frac{\left[i\omega^{\left(j\right)}+\varepsilon +\Sigma_{22}\left(k\right)\right]e^{i\omega^{\left(j\right)}\left(\frac{\mathrm{sign}(\tau )}{2T}-\tau \right)}}{2\left(\omega^{\left(2\right)}-\omega^{\left(1\right)}\right)\sin\left[\omega^{\left(j\right)}/\left(2T\right)\right]},$$

(69) $$G_{12}\left(\tau ,k\right)=\sum_{j=1}^{2}\frac{{\left(-1\right)}^{j}\Sigma_{12}\left(k\right)e^{i\omega^{\left(j\right)}\left(\frac{\mathrm{sign}(\tau )}{2T}-\tau \right)}}{2\left(\omega^{\left(1\right)}-\omega^{\left(2\right)}\right)\sin\left[\omega^{\left(j\right)}/\left(2T\right)\right]},$$

where the two quasiparticle eigen-energies

(70) $$i\omega^{\left(1,2\right)}=\frac{\Sigma_{11}-\Sigma_{22}}{2}\pm {\left[{\left(\varepsilon +\frac{\Sigma_{11}+\Sigma_{22}}{2}\right)}^{2}-\Sigma_{12}\Sigma_{21}\right]}^{\frac{1}{2}}$$

depend on the wave vector k via the self-energies

(71) $$\Sigma_{j_{0}j}\left(k\right)=\sum_{l=0}^{p}\Sigma_{j_{0}}^{j}\left(l\right)e^{-ik(r_{l}-r_{0})},\;r_{l}=r_{0}+l.$$

The latter Fourier transform of the self-energy consists of only 1+p terms within a neighborhood of the nearest sites for which there are nonzero couplings $J_{r_{0},r_{l}}\neq 0$. This is a consequence of the fact that the self-energy matrix is a 2(p+1)-banded matrix. The (2×2)-matrix blocks Σ(l) are given explicitly in Equations (57)–(59) representing the exact solution to Equation (17).

The spatial Fourier transforms of the normal and anomalous equal-time correlation functions follow from Equations (68) and (69) in the limit τ→+0:

(72) $$g_{11}\left(k\right)=\sum_{j=1}^{2}\frac{{\left(-1\right)}^{j}\left[i\omega^{\left(j\right)}+\varepsilon +\Sigma_{22}\left(k\right)\right]}{i\left(\omega^{\left(1\right)}-\omega^{\left(2\right)}\right)\left[1-\exp\left(-i\omega^{\left(j\right)}/T\right)\right]},$$

(73) $$g_{12}\left(k\right)=\sum_{j=1}^{2}\frac{{\left(-1\right)}^{j}\Sigma_{12}\left(k\right)}{i\left(\omega^{\left(2\right)}-\omega^{\left(1\right)}\right)\left[1-\exp\left(-i\omega^{\left(j\right)}/T\right)\right]}.$$

Thus, we derive the equations for the values of the normal and anomalous correlation functions at (1+p) difference position vectors $l=r_{l}-r_{0}$ of the neighboring spins:

(74) $$g_{1j}\left(l\right)={\left(\frac{a}{L}\right)}^{d}\sum_{k}g_{1j}\left(k\right)e^{ikl},\;j=1,2;\;l=0,1,\ldots ,p.$$

Their right hand side is determined by the left hand side $g_{1j}\left(l\right)$ itself via Equations (57) and (70)–(73). They constitute an exact closed system of 2(1+p) self-consistency equations. Its finding is related to a solution of the Ising problem in the same way as finding of a self-consistency equation in the mean-field theory is related to a solution of a phase transition problem. However, now the self-consistency Equation (74) is an exact equation valid in the entire critical region, not just its mean-field approximation. One can analyze these explicit exact self-consistency equations by the well-known in the mean-field theory analytic and numerical tools. It is relatively simple for the Ising model with the zero off-diagonal self-energy $\Sigma_{12}=0$ and zero anomalous correlations, when only the (1+p) self-consistency equations remain. Moreover, in the isotropic case, when the cross-correlations with all *p* nearest neighbors are the same, the system is reduced to just two equations.

Note that the close exact self-consistency equations exist only for the unconstrained, auxiliary basis normal and anomalous auto- and cross-correlations. When the latter are found, the actual, observable statistical and thermodynamic quantities can be explicitly expressed in terms of those basis correlations via the matrix permanent, as is shown in the Section 3.2, Section 3.3 and Section 3.4 for the true, constrained partition function, order parameter, correlation functions, and joined statistics of the spin-boson occupations (in particular, see Equations (31)–(35), (45) and (55)).

This completes the exact general reduction of the Ising model to computing the matrix permanent and provides a basis for the calculation of all statistical and thermodynamic characteristics of the critical phenomena via the permanent of the sub-matrices composed from a relatively simple, unconstrained spin-boson correlation matrix *g*.

Next, we proceed with a discussion of other remarkable features and representations of the matrix permanent ad rem to the analysis of the nature’s complexities.

## 4. The Permanent and the Fractals

Here and in Section 5 we present a remarkable finding of a direct relation between the permanent and the fractals and chaos. It is based on the two new integral representations of the n×n matrix permanent via (i) an analog of the famous Weierstrass function which is known for its fractal structure and nontrivial Hausdorff dimension and (ii) a mean value of a random multivariate polynomial.

We show that the fractals and chaos are intrinsic to evaluation of the matrix permanent.

### 4.1. The 1d Integral Representation of the Permanent: A Fractal Integrand and a Weierstrass Function

A move is to find an integral representation of the permanent for any n×n matrix $A_{pq}$ in a form of a 1d integral. The idea is as follows. Let us form a sum of quasi-harmonics $c_{k}e^{i\pi t\nu_{k}}$ at frequencies $\nu_{k}$ with amplitudes $c_{k}$ determined by the matrix entries $A_{pq}$ in such a way that one spectral component of a known frequency $\nu_{0}$ would have the amplitude equal to the permanent, $c_{0}=\mathrm{per}A$. Then, employ an appropriate Fourier integral to discriminate this component and find the permanent as its amplitude. So, we introduce a permanental function $P_{A}\left(z\right)$ as a product of the row functions $B_{p}\left(z\right)$,

(75) $$P_{A}\left(z\right)=\prod_{p=1}^{n}B_{p}\left(z\right),\;B_{p}\left(z\right)=\sum_{q=1}^{n}A_{pq}z^{b^{q-1}}.$$

The amplitude of the spectral component at the frequency $\nu_{0}=\sum_{q=1}^{n}b^{q-1}=\frac{b^{n}-1}{b-1}$ gives the permanent, $c_{0}=\mathrm{per}A$, for any base b>1 if there are no coincidental resonances: $\sum_{q=1}^{n}n_{q}b^{q-1}\neq \nu_{0}$ for any partition $\left\{n_{q}\geq 0|q=1,\ldots ,n\right\}$ of $n=\sum_{q}n_{q}$ except the unity one, $n_{q}=1\;\forall q$. Under this condition, we find the permanent’s 1d integral representation as follows

(76) $$\begin{array}{cc} \; & \mathrm{per}A=\frac{1}{2}\int_{-1}^{1}{\bar{P}}_{A}\left(e^{i\pi t}\right)dt\;\mathrm{for}\mathrm{an}\mathrm{integer}\mathrm{base}\;b=2,3,\ldots ,\\ \; & \mathrm{per}A=\lim_{T\to \infty }\frac{1}{2T}\int_{-T}^{T}{\bar{P}}_{A}\left(e^{i\pi t}\right)dt\;\mathrm{for}a\mathrm{non}-\mathrm{integer}\mathrm{base}\;b>1. \end{array}$$

Here an integrand is a following function of a complex variable $z=e^{i\pi t}$,

(77) $${\bar{P}}_{A}\left(z\right)=\prod_{p=1}^{n}{\bar{B}}_{p}\left(z\right);\;{\bar{B}}_{p}\left(z\right)=z^{\frac{b^{n}-1}{n(1-b)}}\sum_{q=1}^{n}A_{pq}z^{b^{q-1}}.$$

For an integer base *b*, this function is a polynomial in *z*. Its spectrum $\nu_{k}\in \left[n-\frac{b^{n}-1}{b-1},nb^{n-1}-\frac{b^{n}-1}{b-1}\right]$ is exponentially broad, with big **Hadamard gaps**. It was designed so on purpose to make the 1d representation (76) possible.

At b≥2, Equation (76) remains valid even if the row function $B_{p}$ is extended from a finite sum of n terms to an infinite series ${\tilde{B}}_{p}\left(z\right)=\sum_{q=1}^{\infty }A_{pq}z^{b^{q-1}}$ by adding the higher *z*-powers with q>n and any, unrelated to *A*, factors. The series ${\tilde{B}}_{p}$ is a Weierstrass-type function, like a complex extension

(78) $${\tilde{W}}_{a,b}\left(z\right)=\sum_{k=0}^{\infty }a^{-k}z^{b^{k}},\;z\in C,\;\left|z\right|\leq 1,$$

of the Weierstrass cosine function Wa,b(t)=ReW˜a,b(eiπt). *The Weierstrass functions are famous for being continuous everywhere but differentiable nowhere.* The extension ${\tilde{W}}_{a,b}\left(z\right)$ is a lacunary (cf. Hadamard gaps) complex power series. A fractal (box or, as is believed, Hausdorff) dimension D=2−α of the $W_{a,b}\left(t\right)$ graph is determined by the Hurst, or scaling, exponent $\alpha =\log_{b}a$. The dimension is greater than unity, D>1, if a∈(1,b); see reviews on a fractal geometry of the Weierstrass functions [55,56]. It is known that an image of the unit circle, |z|=1, under the complex-valued Weierstrass map ${\tilde{W}}_{a,b}\left(z=e^{i\theta }\right),\theta \in \left[-\pi ,\pi \right],$ of an integer base *b* covers an open subset of the complex plane, that is, forms *a Peano curve*, if the amplitude *a* is close to 1, that is if the fractal dimension, $D=2-\log_{b}a$, of $W_{a,b}\left(t\right)$ is close to 2. Moreover, in this case the complex-valued map ${\tilde{W}}_{a,b}\left(e^{i\theta }\right),\theta \in \left[-\pi ,\pi \right],$ as a subset in the 3d space {θ,ReW˜a,b,ImW˜a,b}∈R3 is a fractal of the box dimension $D_{3}=2D-1=3-2\log_{b}a$ close to 3 and almost fully fills in an open subset of the 3d space as the 3d Peano curve.

Thus, the result in Equation (76) reveals a fractal nature of the permanent discussed in detail below. For the critical phenomena, in particular, for the Ising model, the matrix $A_{pq}$ is determined by the correlation function of the unconstrained bosons and evolves from a fast exponential decay, for example, $A_{pq}∼b^{-\alpha q}$, in the disordered phase to a slower than exponential, for example, power-law $A_{pq}∼q^{-\eta },\eta >0,$ decay in the ordered phase. In terms of the permanental row functions ${\tilde{B}}_{p}\left(z\right)$, such an evolution of the many-body system across the critical region means a transition from (a) the Weierstrass-type function of a large exponent α≈1 and a trivial, almost non-fractal structure with the dimension D≈1 through (b) a sequence of the Weierstrass-type functions of a smaller exponent α≪1 and a nontrivial fractal structure with the dimension D=2−α larger than unity towards (c) the Weierstrass-type functions of an effectively zero exponent α≈0 and a fully developed fractal structure with the maximal dimension D≈2.

### 4.2. A Fractal Nature of the Matrix Permanent

Here we demonstrate phenomenal fractal properties of the permanental function ${\bar{P}}_{A}\left(z\right)$ forming the 1d integral representation in Equation (76). They manifest themselves already in the cases of very simple n×n matrices $A_{pq}\equiv 1$, $A_{pq}=a^{-q}$, and $A_{pq}=1+a\delta_{p,q}$ who’s permanents, perA, are n!, $n!/a^{n(n+1)/2}$, and $e^{a}\Gamma \left(n+1,a\right)$ (see Equation (142) below), respectively. In the first two cases the permanental function can be replaced by the *n*-th power of the Weierstrass function in Equation (78), ${\tilde{P}}_{A}\left(z\right)={\left[{\tilde{W}}_{a,b}\left(z\right)\right]}^{n}$.

In order to illustrate a fractal nature of the permanent, let us consider the fractal properties of the integrand and the integral’s accumulation in the permanent’s representation (76) with the integration range increasing from zero to the ultimate value T=1 or T=∞ for the integer or non-integer base *b*, respectively:

(79) $$I_{A}\left(T\right)=\frac{1}{2T}\int_{-T}^{T}{\bar{P}}_{A}\left(e^{i\pi t}\right)dt.$$

First, we elaborate on the basic case of the n×n matrix with unity entries, $A_{pq}\equiv 1$, for which the asymptotics of the permanent is given by the Stirling’s formula,

(80) $$\mathrm{per}\left(A_{pq}\equiv 1\right)=n!∼\sqrt{2\pi n}\;\frac{n^{n}}{e^{n}}\;\mathrm{at}\;n\to \infty .$$

We find that there are two qualitatively different patterns by which the integral (79) approaches the permanent’s value: $I_{A}\left(T\right)\to \mathrm{per}A$ at T→1 or *∞*. Which of the two patterns is realized depends on whether the base *b* of the permanental function ${\bar{P}}_{A}$ is less or larger than the base of the exponential factor in the denominator of the permanent’s asymptotics, which is e=2.718… in the case of Equation (80). (A factor $n^{n}$ could be eliminated by re-scaling the matrix *A*, say, to a doubly stochastic one.)

### 4.3. Permanent’s Fractal: The Case of the Integer Base

**Figure 1:**
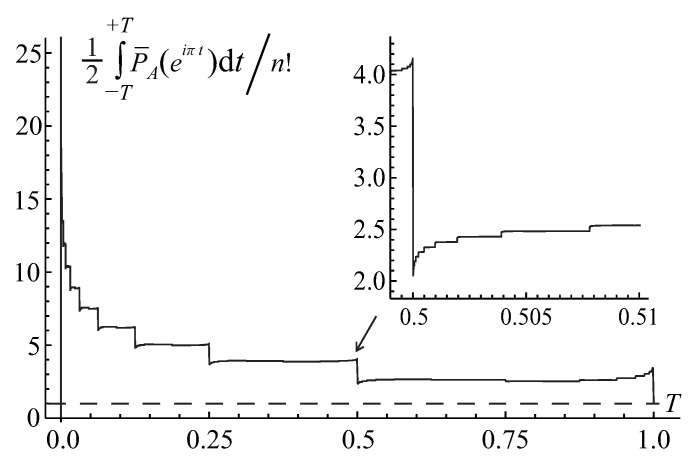
A fractal pattern of an accumulation of the integral in Equation (79) with an increasing range of the integration, [−T,T], for the n×n matrix $A_{pq}=1$ in the case of n=20 and the integer base b=2. At T→1, the integral converges to the permanent’s exact value, perA=n!, in accord with the permanent’s integral representation in Equation (76). The insert magnifies a self-similar fractal structure caused by a hierarchy of extrema of the permanental function ${\bar{P}}_{A}\left(e^{i\pi t}\right)$ shown in Figure 3 in a vicinity of the primary extremum $t_{1}=\frac{1}{2}$.

The first pattern is illustrated in Figure 1 for the binary base b=2<e. In this scenario, the integral in Equation (79) quickly reaches an exceedingly large maximum value ∼$n^{n}/b^{n}\gg \mathrm{per}A$ at an exponentially small displacement $T∼1/b^{n}\ll 1$ from zero and then gradually, with some oscillations, decreases by a sequence of fractal, self-similar steps to the actual value of the permanent as the integration range *T* tends to unity. (Hereafter, for simplicity’s sake, we skip a logarithmic factor ∼$\log_{b}n$ in all of the order-of-magnitude estimates.)

**Figure 2:**
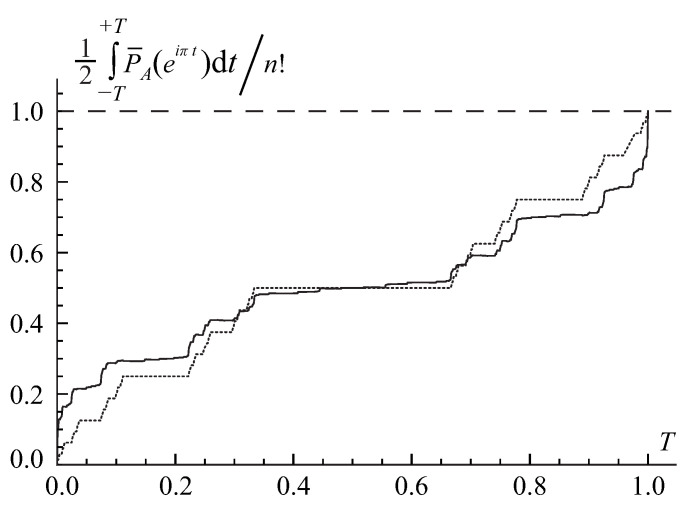
A fractal, similar to a Cantor-Lebesgue function or Devil’s staircase (dotted curve), pattern of the accumulation of the integral in Equation (79) (solid curve) with an increasing range of the integration, [−T,T], for the n×n matrix $A_{pq}=1$ in the case of n=10 and the integer base b=3. At T→1, the integral converges to the exact value of the permanent, perA=n!, as per the permanent’s representation in Equation (76).

The second pattern is illustrated in Figure 2 for the ternary base b=3>e. In this scenario, the integral in Equation (79) gradually grows from zero to the permanent’s value perA all the way from T=0 to T=1. This pattern has a fractal, self-similar structure similar to a famous Cantor-Lebesgue function, or Devil’s staircase [56].

**Figure 3:**
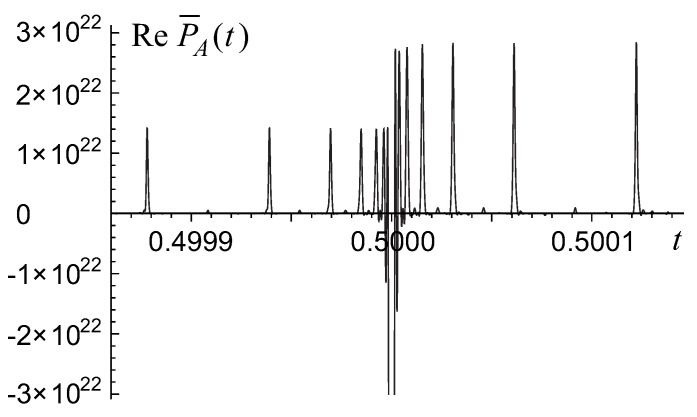
A fractal hierarchy of extrema for the permanental function ReP¯A in Equation (81): Two sequences of peaks of the secondary series of extrema located at $t_{k_{1},k_{2}}^{\left(s_{2}\right)}=\frac{1}{2^{k_{1}}}+\frac{s_{2}}{2^{k_{1}+k_{2}}}$, $k_{2}=1,\ldots ,n-1-k_{1}$, to the left ($s_{2}=-1$) and right ($s_{2}=+1$) from the extremum $k_{1}=1$, $t_{1}=\frac{1}{2}$, of the primary series; cf. Figure 1. The integer base is b=2, matrix size n=20,a=1.

These patterns could be unambiguously understood by taking into account a remarkable fine structure of the permanental function ${\tilde{P}}_{A}={\left[{\tilde{W}}_{a,b}\left(e^{i\pi t}\right)\right]}^{n}$ which is neither a smooth function nor a structureless noise, but a fractal hierarchy of the ultrashort pulses/peaks of a width $\Delta t∼b^{-n}$ and an amplitude regularly scaling from one hierarchy level to the next one. Its fractal structure stems from a more elementary fractal structure of each Weierstrass-function factor. A real part of the function

(81) $${\bar{P}}_{A}=e^{i\pi t\frac{1-b^{n}}{b-1}}{\left[{\tilde{W}}_{a,b}^{\left(n-1\right)}\left(t\right)\right]}^{n},\;{\tilde{W}}_{a,b}^{\left(n\right)}\left(t\right)=\sum_{k=0}^{n}\frac{e^{i\pi tb^{k}}}{a^{k}},$$

involving a truncated version of the Weierstrass function relevant to a matrix of a finite size *n*, is shown in Figure 3.

A primary series of extrema for the permanental function ${\bar{P}}_{A}$ consists of the pulses located near the points $t_{k_{1}}=2^{-k_{1}},k_{1}=0,1,\ldots ,n-1$. At the large matrix size n→∞, all of them, except a few (logarithmic number of) pulses located close to the boundary values of the index $k_{1}$ (say, $k_{1}=0,1,2,3$ and $k_{1}=n-4,n-3,n-2,n-1$), have equal amplitudes and a universal profile F(Δt),

(82) $${\bar{P}}_{A}\left(e^{i\pi (t_{k_{1}}+\Delta t)}\right)\approx \zeta n^{n}F\left(\Delta t\right),\;\zeta =e^{\Delta W(1)-2},$$

localized within a narrow deviation $\Delta t∼2^{-n}$ from the points $t_{k_{1}}$ and described by a special function

(83) $$F\left(\Delta t\right)=e^{\Delta W\left(2^{n}\Delta t\right)-i\pi 2^{n}\Delta t},\;\Delta W\left(y\right)=\sum_{k=1}^{\infty }\left(e^{i\pi y/2^{k}}-1\right).$$

The primary series of extrema is preceded by an exceptionally large, main peak located at the origin t=0 and holding the same universal profile of the width ∼$2^{-n}$,

(84) $${\bar{P}}_{A}\left(e^{i\pi \Delta t}\right)\approx n^{n}F\left(\Delta t\right).$$

A secondary series of extrema for the permanental function ${\bar{P}}_{A}$ consists of two sequences of pulses located to the right ($s_{2}=+1$) and to the left ($s_{2}=-1$) from each primary extremum $t_{k_{1}}$ at the points $t_{k_{1},k_{2}}^{\left(s_{2}\right)}=\frac{1}{2^{k_{1}}}+\frac{s_{2}}{2^{k_{1}+k_{2}}}$; $k_{2}=1,2,\ldots ,n-1-k_{1}$. Namely these two sequences of secondary peaks are shown in Figure 3 in a vicinity of the primary extremum at the point $t_{1}=\frac{1}{2}$ in the case of a=1,b=2,n=20. This fractal, self-similar hierarchy of the enclosed into each other extrema’s series continues with the ternary and higher, *r*-order series of pulses surrounding each (r−1)-order extremum at the points

(85) $$t_{k_{1},k_{2},\ldots ,k_{r}}^{\left(s_{2},\ldots ,s_{r}\right)}=\frac{1}{2^{k_{1}}}+\sum_{j=2}^{r}\frac{s_{j}}{2^{k_{1}+k_{2}+\ldots +k_{j}}}\;;\;k_{j+1}=1,2,\ldots ,n-1-k_{1}-\sum_{m=2}^{j}k_{m}\;,\;s_{j}=\pm 1.$$

At the large matrix size n→∞, all of the *r*-order pulses, except a logarithmic number of pulses located close to the boundary values 1 and $n-1-k_{1}-\sum_{j=2}^{r-1}k_{j}$ of the index $k_{r}$, have equal amplitudes and the universal profile:

(86) $${\bar{P}}_{A}\left(e^{i\pi (t_{k_{1},k_{2},\ldots ,k_{r}}^{\left(s_{2},\ldots ,s_{r}\right)}+\Delta t)}\right)\approx \zeta^{r}n^{n}F\left(\Delta t\right).$$

The extrema’s amplitudes in this hierarchy of series exponentially decrease with increasing order *r* of the series as the *r*-th power of the scaling factor defined in Equation (82),

(87) $$\zeta \approx -e^{-3.39465+i2.48105}\approx -0.026495+i0.020586.$$

At the same time, the number of pulses in the series grows with increasing order *r* of the series roughly as $2^{r-1}C_{n-2}^{r}$, where a binomial coefficient $C_{n-2}^{r}$ accounts for a number of *r*-compositions of the integer $n-1=\sum_{j=1}^{r}k_{j}$ into a sum of *r* integers $k_{j}\geq 1$ and a factor $2^{r-1}$ accounts for a presence of two, right and left, branches of extrema ($s_{j}=\pm 1$) for each *j*-series in the hierarchy.

For a finite matrix size *n*, each pulse contributes to the integral in Equation (76), representing the matrix permanent perA, with the universal factor on the order of the pulse width ∼$2^{-n}$. Thus, the convergence of the integral in Equation (79) to the exact permanent’s value, $I_{A}\left(T\right)\to \mathrm{per}A$, with the increasing range of integration T→1, shown in Figure 1, is a subtle interplay between the effects of decreasing amplitude (∼$\zeta^{r}$) and increasing number (∼$C_{n-2}^{r}$) of pulses in the extrema’s *r*-series as well as a phase modulation of their contribution. The $I_{A}\left(T\right)$ accumulates only the real part of the complex-valued permanental function ${\bar{P}}_{A}\left(e^{i\pi t}\right)$ which contains a phase shift varying with the increasing order *r*, that is evidenced already by the fact that the factor $\zeta^{r}$ in Equation (86) holds the complex number (87). This interplay results in the exponentially small prefactor ∼$\sqrt{2\pi n}\left(2^{n}/e^{n}\right)\ll 1$ in front of the product of the pulse width, $2^{-n}$, and normalization, $n^{n}$, factors in the value of the permanental integral in Equation (79) at T=1, $I_{A}\left(T=1\right)=\mathrm{per}A$, as is required by the Stirling’s formula in Equation (80). This observation explains why for the binary base b=2<e the accumulation pattern of the permanental integral in Equation (79), shown in Figure 1, involves a huge initial growth of the integral to a value ∼$n^{n}/2^{n}$ due to the main peak (84) and, then, its subsequent almost complete cancellation and fine tuning of the interference contributions from many extrema’s *r*-series of different orders *r* which finally (at T=1) lead to the actual, exponentially smaller by the factor ∼$\sqrt{2\pi n}\left(2^{n}/e^{n}\right)\ll 1$, value of the permanent.

A similar analysis could be done for the ternary, b=3>e, and other integer bases larger than *e*. In this case, the width of the pulses in the hierarchy of the extrema’s series is on the order of $b^{-n}$ that is much smaller than the exponential factor $e^{-n}$ required by the Stirling’s formula (80). It yields a value that is much less, by an exponential factor ∼$e^{n}/\left(b^{n}\sqrt{2\pi n}\right)\ll 1$, than the actual value of the permanent and calls for an accumulation of the contributions from many extrema’s *r*-series. This observation explains the fractal pattern of the Cantor-Lebesgue, or Devil’s staircase, type in Figure 2.

These results suggest that, starting from a wide enough range of the integer bases $\left[2,b_{\max}\right]$ and dividing it in halves according to the observed patterns of the permanental integral accumulation shown in Figure 1 or Figure 2, one could find, in a logarithmic number of steps, an approximation of the exponential factor $n^{n}/b_{A}^{n}$ in the permanent’s asymptotics by bounding a true asymptotics’ base $b_{A}$ between the two neighboring integer bases, $b<b_{A}<b+1$, as it was demonstrated above for the case of the Stirling’s asymptotics in Equation (80) where $b=2<b_{A}=e<b+1=3$.

### 4.4. Permanent’s Fractal: The Case of the Non-Integer Base

The analysis presented above could be extended to the non-integer bases by switching to the permanent’s integral representation with the non-integer base b>1 in Equation (76). It constitutes an alternative and, probably, more efficient way of computing the true base $b_{A}$ and the pre-exponential factor in the permanent’s asymptotics, similar to the factor $\sqrt{2\pi n}$ in the Stirling’s asymptotics (80).

For instance, let us illustrate how the permanent’s asymptotics in Equation (80) arises from the integral representation with the natural-logarithm base b=e in Equation (76).

**Figure 4:**
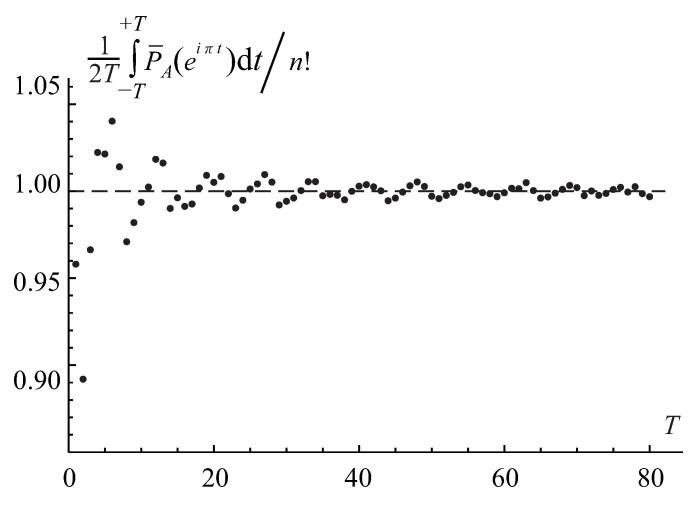
Convergence of the permanent’s integral representation in Equation (76) to the exact value of the permanent, perA=n!, with increasing range of the integration *T* for the n×n matrix $A_{pq}=1$. The circles show the scaled integral in Equation (79) for T=1,…,80. The dashed line corresponds to the exact scaled value, perA/n!, of the permanent. The non-integer base is b=e=2.718…, matrix size n=11.

With increasing range of the integration *T*, it steadily converges to the matrix permanent’s value, as is shown in Figure 4. The analysis of the permanental function with the non-integer base is similar to the binary and ternary ones. Yet, it requires the infinite-limits Fourier integral in Equation (76), instead of the finite-limits integral, for the evaluation of the permanent via the integral spectral discrimination since the permanental function (81) is not a periodical function anymore.

For the case of the n×n matrix with unity entries, $A_{pq}\equiv 1$, the permanental function is given by Equation (81) with the parameters a=1 and b=e as follows

(88) $${\bar{P}}_{A}=e^{i\pi x\frac{1-e^{n}}{e-1}}{\left[{\tilde{W}}_{1,e}^{\left(n-1\right)}\left(t\right)\right]}^{n},\;{\tilde{W}}_{1,e}^{\left(n-1\right)}\left(t\right)=\sum_{q=1}^{n}e^{i\pi tb^{q-1}}.$$

**Figure 5:**
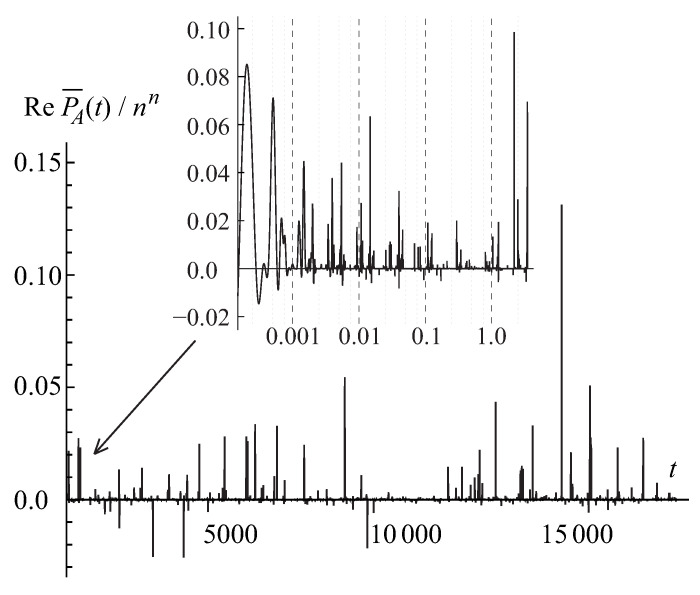
A fractal hierarchy of extrema for the scaled permanental function in Equation (88), ReP¯A(eiπt)/nn. The insert shows the first ten extrema (m=1,…,10) on a logarithmic scale, logt. The base is b=e=2.718…, the matrix size n=10.

**Figure 6:**
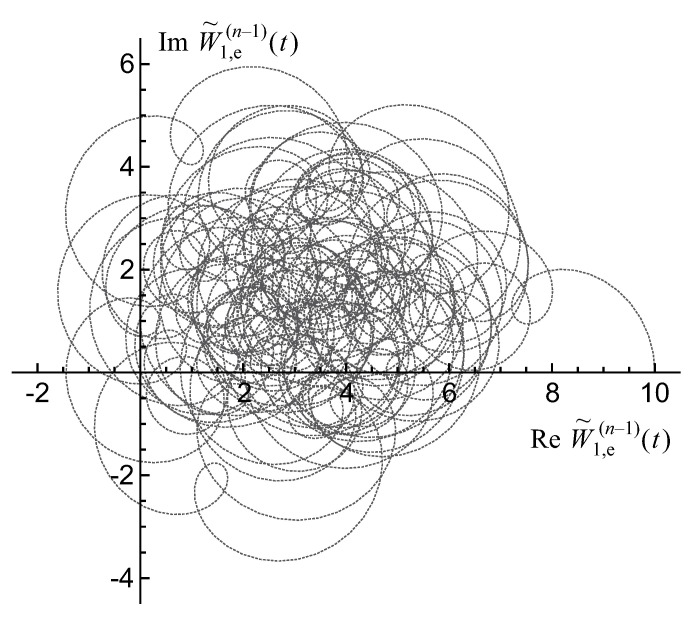
A fractal walk of the permanental row function ${\tilde{W}}_{1,e}^{\left(n-1\right)}\left(t\right)$, Equation (88), on the complex plane starting from its maximum real value ${\tilde{W}}_{1,e}^{\left(n-1\right)}=n$ at t=0. The non-integer base is b=e=2.718…, the matrix size n=10, t∈[0,0.04].

The hierarchy of its extrema is illustrated in Figure 5 and can be understood in terms of a harmonics’ synchronization as follows. Let us consider a differential counter-clockwise rotation of *n* unity-length links (q=1,…,n) in the chain representing the sum, ${\tilde{W}}_{1,e}^{\left(n-1\right)}\left(t\right)$, of *n* harmonics of the row-sum function in Equation (75) on the complex plane of its values. Each subsequent link rotates e=2.718… times faster than the preceding one. The beginning of the chain is rotating about the origin of the complex plane. When the variable *t* runs over the interval t∈[0,T], the end of the chain, that is the row-sum function ${\tilde{W}}_{1,e}^{\left(n-1\right)}\left(t\right)$, follows a path representing its fractal walk on the complex plane shown in Figure 6.

The m=0 extremum is located at the point $t_{0}=0$ where all links (harmonics) have the same orientation in the east direction: $\exp(i\pi t_{0}e^{q-1})=1;\;q=1,\ldots ,n$.

The next, m=1, extremum sits at the point $t_{1}$ where the last, q=n, link (harmonic) makes a full 2π rotation plus a little extra rotation at an angle equal to a total rotation of all other links (harmonics) q=1,…,n−1. The latter condition can be approximately written as a transcendental equation for the position $t_{1}$ as follows

(89) $$\pi t_{1}=2\pi +\arctan\frac{\sum_{q=1}^{n}\sin\left[\pi t_{1}e^{q-1}\right]}{\sum_{q=1}^{n}\cos\left[\pi t_{1}e^{q-1}\right]}\;.$$

For the case of n=10 in Figure 5, Equation (89) gives the value $t_{1}\approx 0.000257$ which is very close to the exact position $t_{1}=0.000271$ of this extremum of the row-sum polynomial in Equation (75). At the point $t=t_{1}$, the next to the last link (harmonic) q=n−1 makes $90^{o}+55^{o}$ degrees, that is about 2π/e, of a counter-clockwise rotation and is directed mainly to the west, while all other links (harmonics) are still directed mainly to the east at moderate to small angles above the positive real-valued axis on the complex plane: 35^o^ for q=n, 53^o^ for q=n−2, 20^o^ for q=n−3, 7^o^ for q=n−4, etc.

The other extrema in the primary series located at the positions $t_{m}<1$, m=2,…,n−1, can be viewed and found similarly. Their positions can be approximated as

(90) $$t_{m}\approx t_{1}e^{m-1}.$$

All these extrema at $t=t_{m},\;m=1,\ldots ,n-1,$ correspond to the configurations with just one link (harmonic of the row-sum polynomial), namely the one labeled by the index q=n−m, directed mainly to the west and all of the other links (harmonics) directed mainly to the east.

The next extremum, m=n, the first one located at the position, $t_{n}\approx t_{1}e^{n-1}>1$, further away from the origin than unity, has all links (harmonics) directed mainly to the east. The following, m=n+1, extremum in the series (90) has only one, namely the last one with q=n, link (harmonic) directed mainly to the west, while all other links (harmonics) are east directed. The next three, m=n+2,n+3,n+4, extrema in the series (90) have only two (q=2n−m+1 and q=2n−m+2) links (harmonics) oriented mainly in the west direction.

This pattern continues in the series (90) as following. The next three, m=n+5,n+6,n+7, extrema have already three (q=2n−m+1,2n−m+2, and q=2n−m+5) links (harmonics) oriented mainly in the west direction. The next two, m=n+8,n+9, extrema have already four (q=2n−m+1,2n−m+2,2n−m+5,2n−m+7) links (harmonics) oriented mainly in the west direction. The next two, m=n+10,n+11, extrema have already five links (harmonics) oriented mainly in the west direction. Then the pattern becomes more complicated but still can be followed via the picture of differential rotation of links in the chain of harmonics. Of course, a similar picture is valid for any *n*, not only for n=10.

Obviously, there are many higher-order series of extrema in the fractal hierarchy of extrema of the permanental function (88). All of them correspond to the local extrema of the row-sum polynomial in Equation (75), that is, the function ${\tilde{W}}_{1,e}^{\left(n-1\right)}\left(t\right)$. In fact, at large *n*, there exist exponentially many closely located local extrema of the row polynomial that makes extremely difficult to numerically differentiate a particular *m*-order extremum from other series’ extrema. Such a Weierstrass fractal structure is globally homogeneous, ergodic along the t axis since this property is required for the asymptotically linear growth of the permanental integral in Equation (76) with an increasing range limit T→∞.

We find that, with increasing range of the variable t∈[−T,T],T→∞, the path of the scaled first-row permanental function for the n×n matrix $A_{pq}=1$,

(91) $$\frac{{\bar{B}}_{1}\left(e^{i\pi t}\right)}{A_{1}}=\frac{e^{i\pi t\frac{e^{n}-1}{n(1-e)}}}{A_{1}}\sum_{q=1}^{n}A_{1q}e^{i\pi te^{q-1}},\;A_{1}=\sum_{q=1}^{n}A_{1q},$$

introduced in Equation (75) and additionally scaled by its maximum value *n*, fully covers a finite 2d region in the complex plane enclosed by a hypocycloid,

(92) $$z\left(\theta \right)=\frac{n-1}{n}e^{i\theta }\left(1+\frac{1}{n}e^{-in\theta }\right),\;\theta \in \left[0,2\pi \right].$$

A number of its cusps is equal to the size *n* of the matrix. Remarkably, in the limit n→∞, the hypocycloid perimeter, $P_{n}$, remains longer than the unit circle perimeter, 2π, by a finite amount,

(93) $$P_{n}=8\left(1-\frac{1}{n}\right)\to 8>2\pi ,$$

although its area, $S_{n}$, tends to the area of the unit circle,

(94) $$S_{n}=\pi \left(1-\frac{1}{n}\right)\left(1-\frac{2}{n}\right)\to \pi .$$

So, the related scaled permanental function entering the permanent’s integral representation in Equation (76), ${\bar{P}}_{A}/n^{n}={\left[{\bar{B}}_{1}\left(e^{i\pi t}\right)/n\right]}^{n}$, fully covers a finite 2d region in the complex plane enclosed by the *n*-th power of the hypocycloid that acquires a teardrop shape at n≫1,

(95) $${\left[z\left(\theta \right)\right]}^{n}\to \frac{1}{e}\left(\frac{1}{n-1}+e^{i\theta }\right)e^{e^{-i\theta }}\;\mathrm{at}\;n\gg 1,\;\theta \in \left[0,2\pi \right].$$

**Figure 7:**
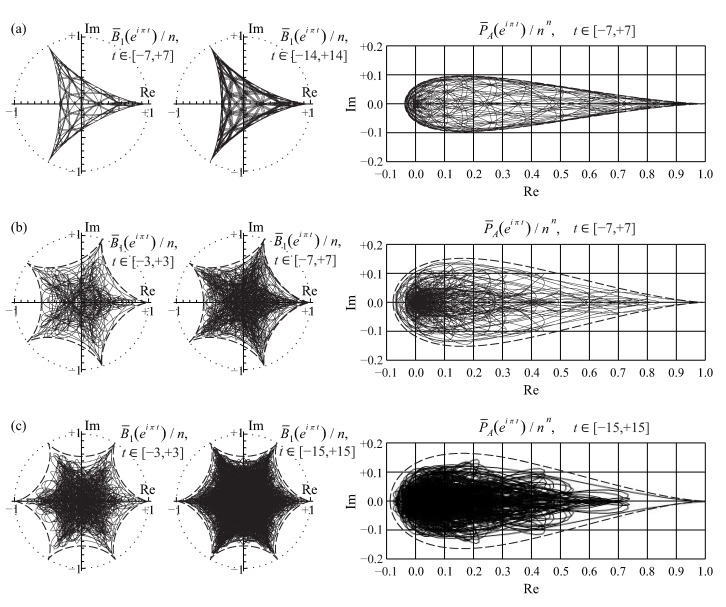
A fractal walk of the scaled row-sum function (91) (the two left insets) and the scaled permanental function, ${\bar{P}}_{A}/n^{n}={\left[{\bar{B}}_{1}\left(e^{i\pi t}\right)/n\right]}^{n}$, (the right insert) on the complex plane with the independent variable *t* running over an indicated interval t∈[−T,T] for the matrix $A_{pq}=1$ of the sizes n=3 (**a**), n=5 (**b**) and n=6 (**c**). The fractal support’s border is the hypocycloid (92) with *n* cusps enclosed with the unit circle. With increasing range of walk, T→∞, the fractal fully covers the support. The base is b=e=2.718….

This fractal property of the row and entire permanental functions is reminiscent of the properties of the Peano and similar fractal curves and illustrated in Figure 7.

**Figure 8:**
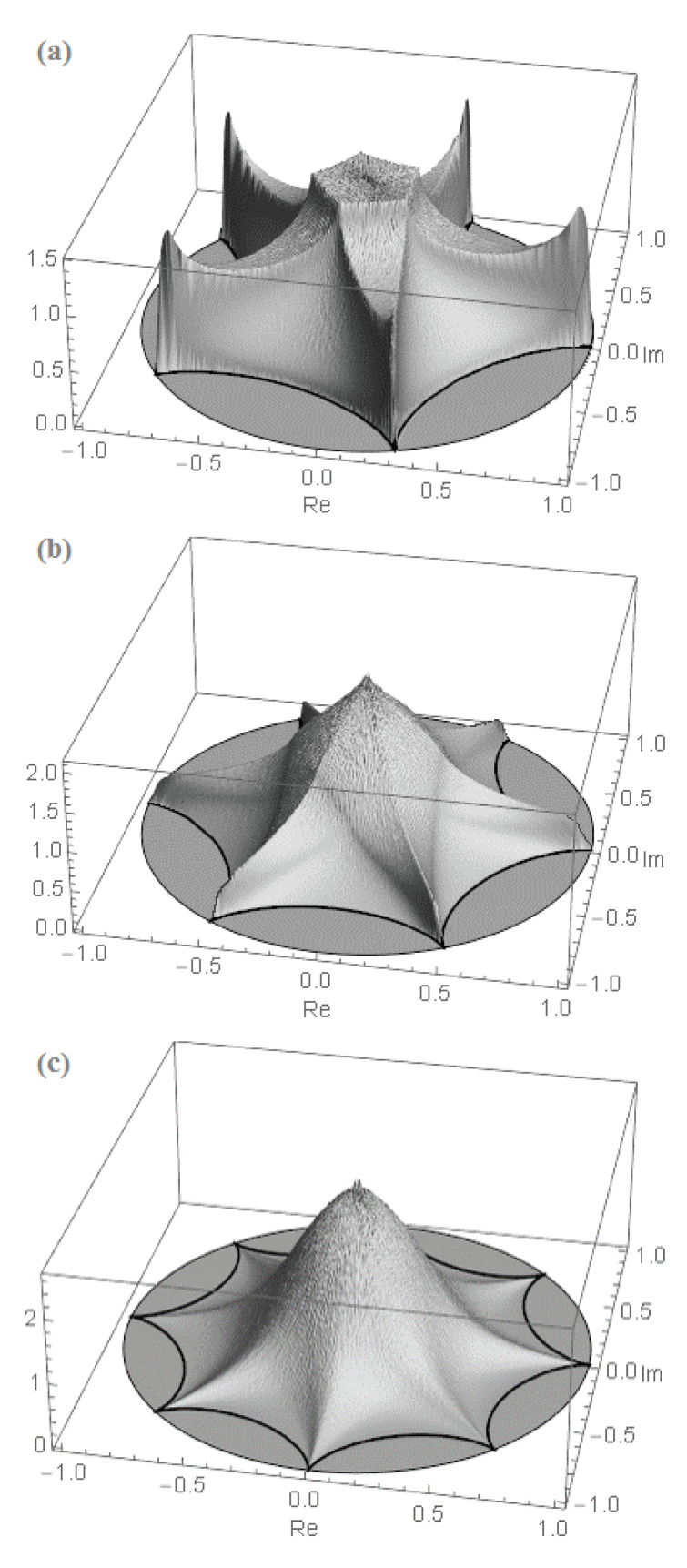
The 2d probability density function $\rho_{1}^{\left(p\right)}\left(u,v\right)$ of the fractal row function in Equation (91) for the n×n matrix $A_{pq}=1$ with n=5 (**a**), n=6 (**b**) and n=8 (**c**). The border of the fractal is clearly visible as the hypocycloid (92) enclosed with the unit circle. The non-integer base is b=e=2.718….

**Figure 9:**
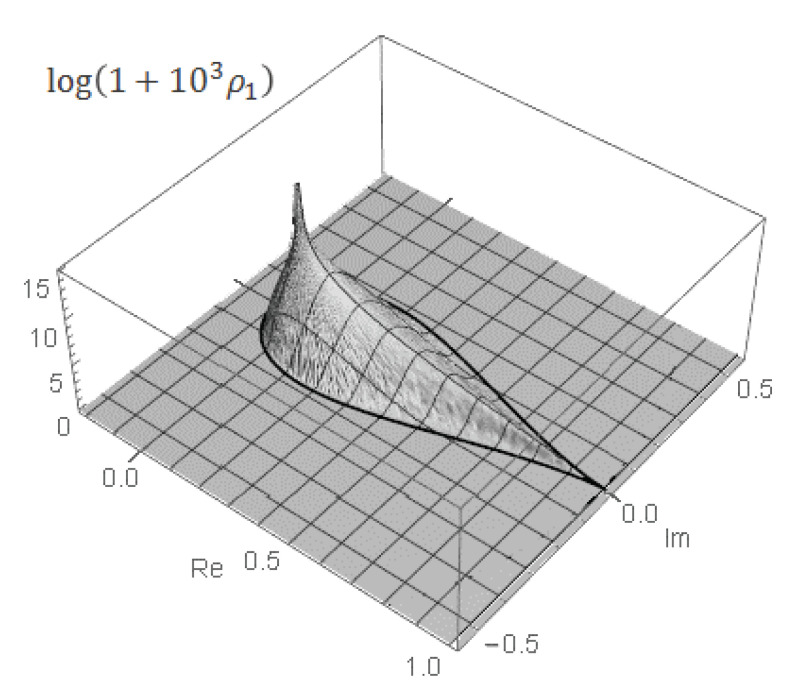
A scaled logarithm, $\log(1+10^{3}\rho_{1})$, of the 2d probability density function $\rho_{1}\left(u,v\right)$ of the fractal permanental function in Equation (76), ${\bar{P}}_{A}/n^{n}$, for the n×n matrix $A_{pq}=1$. The base is b=e=2.718…, the matrix size n=8.

As a result, the row and entire permanental functions, ${\bar{B}}_{p}$ and ${\bar{P}}_{A}$, possess a nontrivial ergodic fractal measures (i.e., 2d probability density functions $\rho_{1}^{\left(p\right)}\left(u,v\right)$ and $\rho_{1}\left(u,v\right)$, respectively) with a support on the aforementioned finite region of the complex plane z=u+iv. For the row function of the matrix $A_{pq}=1$, it is shown in Figure 8 for the matrix size n=5,6,8. The distribution function $\rho_{1}\left(u,v\right)$ for the entire permanental function is shown in Figure 9.

The analysis of the fractal properties of the permanental functions entering the permanent’s integral representation in Equation (76), that was presented above for the case of the matrix $A_{pq}=1$, could be easily extended to the matrices $A_{pq}=a^{-q}$ and $A_{pq}=1+a\delta_{p,q}$, who’s permanents $n!/a^{n(n+1)/2}$ and $e^{a}\Gamma \left(n+1,a\right)$, as per Equation (142), are known, as well as to other circulant matrices with still unknown permanents. We skip it here.

**Figure 10:**
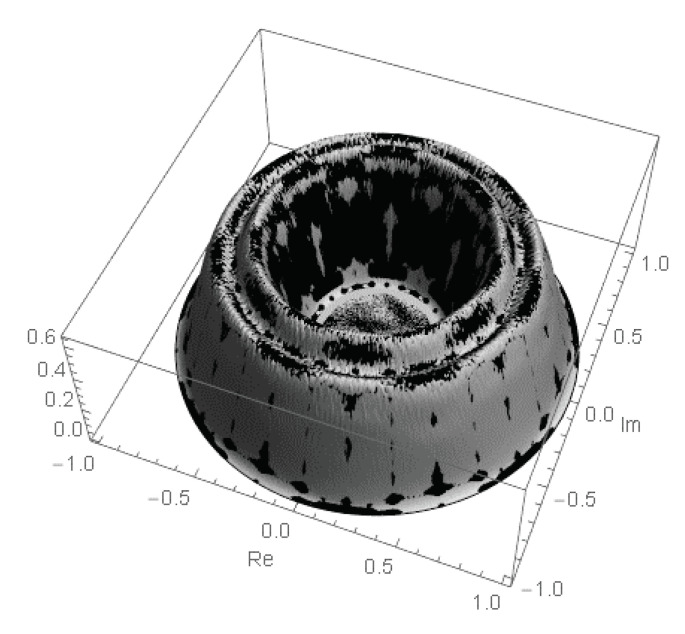
The 2d probability density function $\rho_{1}^{\left(1\right)}\left(u,v\right)$ of the scaled fractal first-row function (91), ${\bar{B}}_{1}/A_{1}$, (gray contour) closely reproduced by the zeroth-order approximation (104) of its multivariate counterpart $\rho_{n}^{\left(1\right)}\left(u,v\right)$ (black contour) for the n×n circulant matrix with a power-law decay of its first-row entries, $A_{1q}=q^{-2}$; n=7. The base is b=e=2.718….

We only state that the related row and entire permanental functions possess the similar fractal properties and point to some of their typical modifications due to a variation of the matrix entries. For instance, Figure 10 shows the 2d probability density function $\rho_{1}^{\left(1\right)}\left(u,v\right)$ of the scaled fractal first-row function (91), ${\bar{B}}_{1}/A_{1}$, for the n×n circulant matrix with a power-law decay of its first-row entries, $A_{1q}=q^{-2}$, and n=7. A comparison with Figure 8 clearly proves that the aforementioned fractal behavior in the present case of the circulant matrix with varying entries qualitatively remains the same as in the case of the constant matrix $A_{pq}=1$ discussed above. Just its sharp features become smoother, that is, less pronounced, and the borders and topology of the fractal support region on the complex plane are modified. In particular, an increasing variation of the matrix entries results in an appearance of a no-support, empty region emerging in the central part of the fractal’s support as is shown in Figure 10 and also in Figure 12 below (cf. Figure 7). The inner and outer borders of the permanent’s fractal support could be found analytically, via a method of the Lagrange multipliers, for any complex matrix *A*. The related results will be presented elsewhere.

**Figure 11:**
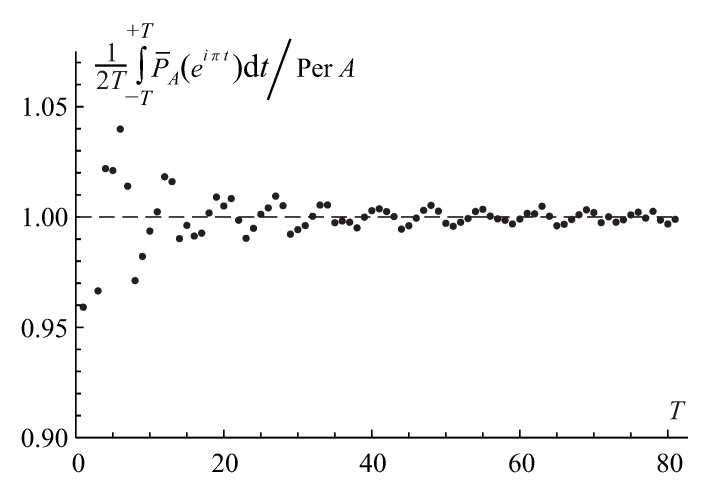
A steady convergence of the permanent’s integral representation in Equation (76) to the exact value of the permanent, $\mathrm{per}A=e^{a}\Gamma \left(n+1,a\right)$, with increasing range of the integration *T* for the circulant n×n matrix $A_{pq}=1+a\delta_{p,q}$: The scaled integral in Equation (79), $I_{A}\left(T\right)/\mathrm{per}A$, as a function of *T*. The base is b=e=2.718…, the matrix size n=11, a=1.

At last, we illustrate a convergence of the integral representation in Equation (76) to the exact value of the permanent in Equation (142), $\mathrm{per}A=e^{a}\Gamma \left(n+1,a\right)$, with increasing range of the integration *T* for the circulant n×n matrix $A_{pq}=1+a\delta_{p,q}$ with varying entries by plotting the relative value of the integral in Equation (79), $I_{A}\left(T\right)/\mathrm{per}A$, with the base b=e=2.718… as a function of *T* in Figure 11. The convergence is stable, similar to that in Figure 4.

## 5. Multivariate Representations of the Matrix Permanent

Here we find a relation of the permanent to complex random polynomials and determinants of many variables.

### 5.1. The Integral Representation of the Permanent via a Multivariate Polynomial of Complex Variables

(96) $$\mathrm{per}A=\int_{-\pi }^{\pi }\ldots \int_{-\pi }^{\pi }{\bar{P}}_{A}\left(\left\{z_{q}\right\}\right)\prod_{k=1}^{n}\frac{d\theta_{k}}{2\pi },\;{\bar{P}}_{A}=\prod_{p=1}^{n}{\bar{B}}_{p}\left(\left\{z_{q}\right\}\right).$$

Here ${\bar{B}}_{p}\left(\left\{z_{q}\right\}\right)=\frac{1}{\tilde{z}}\sum_{q=1}^{n}A_{pq}z_{q},\;z_{q}=e^{i\theta_{q}},\;{\tilde{z}}^{n}=\prod_{q}z_{q}.$ A validity of the formula (96) stems from the construction of the permanental function ${\bar{P}}_{A}$ in such a way that all the terms in Equation (96) which are present in the perA do not depend on the phases $\theta_{q}$ in virtue of the imposed phase-locking factor $\prod_{q=1}^{n}\frac{z_{q}}{\tilde{z}}=1$ and, hence, are not affected by the integration. All the other terms contain, at least, one factor $z_{q}=e^{i\theta_{q}}$ and, hence, vanish after the integration.

### 5.2. Discrete Analogs of the Permanent’s Integral Representations: BBFG Formula & Its Generalization

The method that we employed for the derivation of the permanent’s integral representations in Equations (76) and (96) immediately yields also their discrete analog—a formula of Balasubramanian [57], Bax–Franklin [58] & Glynn [23]

(97) $$\mathrm{per}A=\sum_{\left\{\delta_{q}=\pm 1|q\neq n\right\}}\frac{\prod_{k=1}^{n}\delta_{k}}{2^{n-1}}\prod_{p=1}^{n}\sum_{q=1}^{n}\delta_{q}A_{pq},$$

where $\delta_{q=n}=1$. Namely, it suffices to replace the complex variables $z_{q}=e^{i\theta_{q}}$ running over the unity circle in Equation (96) by the discrete variables $\delta_{q}$ running over two values ±1 and then, instead of imposing the condition $\prod_{q=1}^{n}\frac{z_{q}}{\tilde{z}}=1$ that selects all summands constituting the permanent to be independent on the variables $z_{q}$ and hence not to vanish after applying the integration, multiply the product of the matrix-row sums by the factor $\prod_{k=1}^{n}\delta_{k}$. The latter does the same job of selecting all summands constituting the permanent to be sign independent on the discrete variables $\delta_{q}=\pm 1$, while making any summand irrelevant to permanent the antisymmetric function of those variables $\delta_{q^{\prime }}=\pm 1$ the column’s index $q^{\prime }$ of which is missing in that summand. As a result, the summation $\sum_{\left\{\delta_{q}\right\}}$ in Equation (97) nullifies all these irrelevant summands and yields the exact value of the permanent.

The proof of Equation (97) presented above immediately allows us to conclude that there is an entire series of different discrete-sum representations of the permanent

(98) $$\mathrm{per}A=\sum_{q=1}^{n-1}\sum_{\delta_{q}\in R_{m}}\frac{\prod_{k=1}^{n}\delta_{k}^{m-1}}{m^{n-1}}\prod_{p=1}^{n}\sum_{q^{\prime }=1}^{n}\delta_{q^{\prime }}A_{pq^{\prime }}$$

for any integer m=2,3,…, where the variables $\delta_{q}$ run over the set $R_{m}$ of all *m*-th roots of unity, that is $\delta_{q}^{m}=1$ but $\sum_{\delta_{q}\in R_{m}}\delta_{q}=0$. The BBFG formula (97) is a particular case of the result in Equation (98) at m=2.

### 5.3. Permanent vs. Determinant: The MacMahon Master Theorem

Note that we employed in Section 2 another novel representation of the permanent – via a determinant and the MacMahon master theorem. Here it is given in the integral and discrete forms.

Namely, we can rewrite a well-known MacMahon master theorem [54] as an integral representation of the permanent of any matrix *A* via a n-dimensional Fourier integral

(99) $$\mathrm{per}A=\frac{1}{{\left(2\pi \right)}^{n}}\int_{-\pi }^{\pi }\ldots \int_{-\pi }^{\pi }\frac{\prod_{k=1}^{n}e^{-i\theta_{k}}d\theta_{k}}{\det(1-Az)}\;,$$

where $z=\mathrm{diag}\{z_{k}|k=1,\ldots ,n\}$ is a diagonal matrix with the entries $z_{k}=e^{i\theta_{k}}$. The discrete sum representation is

(100) $$\mathrm{per}A=\lim_{x\to 0}\frac{1}{{\left(2x\right)}^{n}}\sum_{\left\{\delta_{q}=\pm 1|q=1,\ldots ,n\right\}}\frac{1}{\det(\delta -xA)},$$

where the diagonal matrix $\delta =\mathrm{diag}\{\delta_{q}|q=1,\ldots ,n\}$ has the entries $\delta_{pq}=\delta_{q}\delta_{p,q}$ determined by a set of the integers $\left\{\delta_{q}=\pm 1|q=1,\ldots ,n\right\}$ and the Kronecker delta $\delta_{p,q}$. The result (100) stems from the MacMahon master theorem written in a form of the n-dimensional partial derivative

(101) $$\mathrm{per}A=\frac{\partial^{n}}{\partial z_{1}\ldots \partial z_{n}}\frac{1}{\det(1-Az)}{|}_{\left\{z_{k}=0|k=1,\ldots ,n\right\}}$$

taken at the zero values of the variables $z_{k},k=1,\ldots ,n$.

### 5.4. Permanent’s Fractal vs. Complex Stochastic Multivariate Polynomial

The chaotic fractal behavior of the permanental function can be analyzed and understood via the multivariate polynomial of the complex variables comprising the permanent’s integral representation in Equation (96). Here we illustrate this method by two examples.

First, we show that the support region of the fractal associated with the row function in Equation (75) (cf. Equation (91)) on the complex plane is given by a range of the row-sum multivariate polynomial ${\bar{B}}_{p}\left(\left\{z_{q}|q=1,\ldots ,n\right\}\right)$ in Equation (96) for any n×n matrix $A_{pq}$. The row polynomial is a complex-valued stochastic variable given by the function of *n* random phases $\theta_{q},\;q=1,\ldots ,n$, uniformly distributed over their domains $\theta_{q}\in \left[-\pi ,\pi \right]$,

(102) $${\bar{B}}_{p}\left(\left\{z_{q}\right\}\right)=\frac{1}{\tilde{z}}\sum_{q=1}^{n}A_{pq}z_{q},\;z_{q}=e^{i\theta_{q}},\;{\tilde{z}}^{n}=\prod_{q=1}^{n}z_{q}.$$

**Figure 12:**
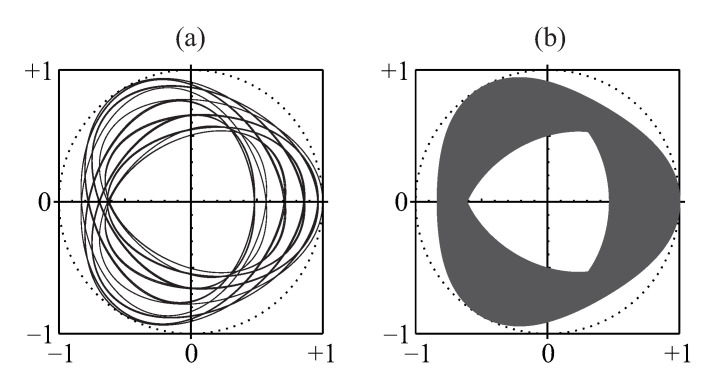
(**a**) A fractal walk of the scaled row-sum function (91) with the independent variable running over the interval t∈[−T,T],T=7, and (**b**) the range of the scaled row-sum multivariate polynomial ${\bar{B}}_{1}\left(\left\{z_{q}|q=1,\ldots ,n\right\}\right)/\left(\sum_{q=1}^{n}A_{1q}\right)$ in Equation (96) on the complex plane for a circulant matrix with the power-law varying entries of the first row $A_{1q}=q^{-2}$. The base is b=e=2.718…, matrix size n=3.

A fact is that mapping the *n*-dimensional domain of variation of a multivariate random vector $\left\{\theta_{1},\ldots ,\theta_{n}\right\}$, that is, the *n*-dimensional direct sum of the *n* intervals [−π,π], or ${\left[-\pi ,\pi \right]}^{n}$, to the complex plane via the row function ${\bar{B}}_{p}\left(\left\{z_{q}\right\}\right)$ in Equation (102) yields the support of the fractal ${\bar{B}}_{p}\left(e^{i\pi t}\right)$ in Equation (77), which is a function of just one real variable *t*. This is shown in Figure 12.

Second, it is possible to find the ergodic measure of the permanental-function fractal in Equation (77) by calculating the 2d probability density function, $\rho_{n}\left(u,v\right)$, of the multivariate polynomial in Equation (96), ${\bar{P}}_{A}\left(\left\{z_{q}\right\}\right)=\prod_{p=1}^{n}{\bar{B}}_{p}\left(\left\{z_{q}\right\}\right)$. The latter can be found by mapping a distribution of the product of *n* random row functions ${\bar{B}}_{p}\left(\left\{z_{q}\right\}\right),\;p=1,\ldots ,n$, from the uniform distribution of the multivariate random phase vector $\left\{\theta_{1},\ldots ,\theta_{n}\right\}$ in the *n*-dimensional cube ${\left[-\pi ,\pi \right]}^{n}$ to the complex plane of the values of ${\bar{P}}_{A}\left(\left\{z_{q}\right\}\right)=u+iv$. For instance, for a n×n matrix *A* of a large size n≫1 and a moderate variation of entries, we find its reasonable zeroth-order approximation analytically as follows

(103) $$\rho_{n}\left(u,v\right)\approx \int_{0}^{\infty }\frac{RJ_{0}\left(Rr^{1/n}\right)}{2\pi nr^{2-2/n}}\prod_{q=1}^{n}J_{0}\left(A_{pq}R\right)\;dR.$$

It is obtained as a distribution function of the *n*-th power of one row function ${\bar{B}}_{1}\left(\left\{z_{q}\right\}\right)$ by neglecting correlations between different row functions ${\bar{B}}_{p}\left(\left\{z_{q}\right\}\right)$ as well as correlations imposed by the phase factor ${\tilde{z}}^{n}$. The zeroth-order approximation for the distribution function of the row function ${\bar{B}}_{p}\left(\left\{z_{q}\right\}\right)$ is

(104) $$\rho_{n}^{\left(p\right)}\left(u,v\right)\approx \frac{1}{2\pi }\int_{0}^{\infty }RJ_{0}\left(Rr\right)\prod_{q=1}^{n}J_{0}\left(A_{pq}R\right)\;dR.$$

**Figure 13:**
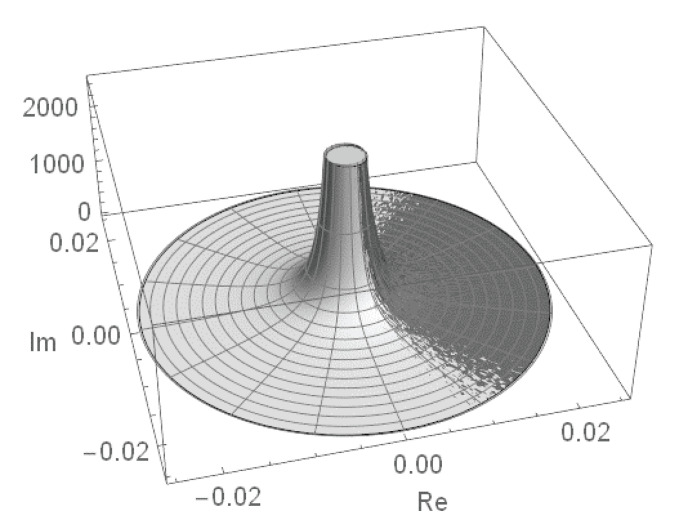
Equal each other distribution functions, $\rho_{1}\left(u,v\right)=\rho_{n}\left(u,v\right)$, of the scaled fractal permanental function in Equation (76), ${\bar{P}}_{A}\left(e^{i\pi t}\right)/A_{1}^{n}$, and scaled complex multivariate polynomial in Equation (96), ${\bar{P}}_{A}\left(\left\{z_{q}|q=1,\ldots ,n\right\}\right)/A_{1}^{n}$, (dark gray) as well as their zeroth-order approximation in Equation (103) (light gray) for the circulant n×n matrix with a varying first row $A_{1q}=q^{-1/2}$; n=8. The base is b=e=2.718….

Here $J_{0}$ is a Bessel function, $r=\sqrt{u^{2}+v^{2}}$. Equations (104) and (103) approximate the main, independent on a polar angle, part of the distributions $\rho_{n}^{\left(p\right)}\left(u,v\right)$ and $\rho_{n}\left(u,v\right)$ quite well. They are shown in Figure 10 and Figure 13 along with the 2d probability density functions $\rho_{1}^{\left(p\right)}\left(u,v\right)$ and $\rho_{1}\left(u,v\right)$ of the fractal row and entire permanental functions in Equation (76), ${\bar{B}}_{p}\left(e^{i\pi t}\right)$ and ${\bar{P}}_{A}\left(e^{i\pi t}\right)$, respectively. In the left three quarters of Figure 13, the approximation (103) (light gray) is a bit larger and, hence, shields the actual distribution (dark gray). In the right quarter, the actual distribution is larger and shields the surface (103). Importantly, we verified that the fractal’s distributions $\rho_{1}^{\left(p\right)}\left(u,v\right)$ and $\rho_{1}\left(u,v\right)$ coincide with the distributions $\rho_{n}^{\left(p\right)}\left(u,v\right)$ and $\rho_{n}\left(u,v\right)$ of the multivariate row and entire permanental functions in Equation (96), ${\bar{B}}_{p}\left(\left\{z_{q}\right\}\right)$ and ${\bar{P}}_{A}\left(\left\{z_{q}\right\}\right)$, respectively.

Thus, the matrix permanent could be calculated as a mean value of the stochastic permanental function or polynomial by averaging their values over the complex plane with the weight given by the distribution function $\rho_{1}\left(u,v\right)$ or $\rho_{n}\left(u,v\right)$, respectively:

(105) $$\mathrm{per}A=\int \left(u+iv\right)\rho_{1}\left(u,v\right)dudv=\int \left(u+iv\right)\rho_{n}\left(u,v\right)dudv.$$

So, finding the next-to-zeroth and higher-order approximations for the distribution function $\rho_{1}\left(u,v\right)$ of the permanent’s fractal for the 1d integral representation in Equation (76) or $\rho_{n}\left(u,v\right)$ for the permanent’s *n*-dimensional integral representation in Equation (96) allows one to compute the permanent via Equation (105).

## 6. Manifestation of a Number-Theoretic Complexity in the Permanent of Schur/Fourier Matrices

Let us relate the complexity of the critical phenomena in physics of many-body systems to the number-theoretic complexity in mathematics via the permanent of a n×n Schur matrix $S_{pq}=e^{2\pi ipq/n}$, employed in a fast Fourier transform, or the degenerate Schur matrices $S_{\nu }$, which differ from *S* by a replication or deletion of some columns.

### 6.1. Permanent’s Representation via Laplace Integrals

Applying the Binet-Cuachy expansion to a circulant n×n matrix $A=PS\Lambda S^{†}P^{-1}/n$, like the one in Equation (2), written via the diagonal matrices of phases $P=\mathrm{diag}(e^{2\pi ip/n})$ and A’s eigenvalues $\Lambda =\mathrm{diag}(\lambda_{p})$, p=1,…,n, we find [59] the permanent of its any (n−m)×(n−m) submatrix ${\left[A\right]}_{\left\{i_{k}\right\}}$ via a sum over the related degenerate Schur submatrices $S_{\nu \{i_{k}\}}$,

(106) $$\mathrm{per}{\left[A\right]}_{\left\{i_{k}\right\}}=\sum_{\nu }\frac{\lambda_{1}^{\nu_{1}}\ldots \lambda_{n}^{\nu_{n}}}{\nu_{1}!\ldots \nu_{n}!}\frac{|\mathrm{per}S_{\nu \{i_{k}\}}{|}^{2}}{n^{n-m}},\;\sum_{j=1}^{n}\nu_{j}=n-m.$$

For the $\mathrm{per}S_{\nu }$, we get the Laplace integral representation

(107) $$\mathrm{per}S_{\nu }=\eta \sum_{Q=1}^{Q_{\max}}\int_{0}^{\infty }\sum_{m_{1},\ldots ,m_{Q}=1}^{n}{\tilde{f}}_{Q}\left(\left\{q_{k},m_{k}\right\}\right)\prod_{k=1}^{Q}\frac{e^{-q_{k}}dq_{k}}{q_{k}},$$

where a coefficient η and a polynomial ${\tilde{f}}_{Q}$ of *Q* variables $q_{k}$ and integers $m_{k}$(k=1,…,Q) defined via a generalized multivariate *q*-Pochhammer symbol [60,61] are specified right after Equation (110).

The results (106) and (107) yield many links between the number theory and permanents. Say, a number of the degenerate Schur matrices with nonzero permanent and a number of terms in the permanent of the generic circulant matrix, both equal $\sum_{d|n}\varphi \left(\frac{n}{d}\right)\frac{(2d-1)!}{d!(d-1)!n}$, are related to the Euler’s totient function φ(k). The permanent of the Schur matrix of the odd order *n* [62], $\mathrm{per}S=\sum_{d|n}u_{n}\left(d\right)\mu \left(\frac{n}{d}\right)$, is related to the M$\ddot{o}$bius function μ(k) and a cardinality $u_{n}\left(d\right)$ of a subset of permutations $\left\{\pi \in S_{n}|\sum_{l=1}^{n}l\pi \left(l\right)=d\;\mathrm{mod}\;n\right\}$. A contribution of the first two orders (Q=1,2) in Equation (107) to this permanent, $\mathrm{per}S=n\left(n-2\right)!\left(H_{n-2}-2+D_{n}\right)+\left\{\mathrm{terms}\;\mathrm{of}\;\mathrm{order}\;Q\geq 3\right\}$, includes the harmonic number $H_{m}=\sum_{k=1}^{m}1/k$ and another number-theoretic function $D_{n}$ involving the greatest common divisor (n,m). The latter is related to the Euler’s totient function and Ramanujan’s sum via a discrete Fourier transform. At last, Equation (107) is equivalent to a sum over multiset partitions, a major focus of the number theory and combinatorics.

More links between the number theory and the permanent could come from Equation (98) which, like the M$\ddot{o}$bius function and the Ramanujan’s sum, involves a sum over the roots of unity.

Thus, the complexity of the major number-theoretic functions is closely related to the complexity of the permanent.

### 6.2. The Permanent of the Schur/Fourier and Circulant Matrices vs. the Number Theory

Here we brief on the details of the rigorous definitions and properties of the circulant and Schur/Fourier matrices, polynomials and number-theoretic functions relevant to the permanent’s representations in Equations (106) and (107).

The n×n circulant matrix *A* with the *p*-th row and *q*-th column entries $A_{pq}$ is a Toeplitz matrix [51] with rows obtained via the consecutive cyclic permutations of the elements of the first row. It is given by the discrete Fourier transform of the set of its eigenvalues $\left\{\lambda_{l}|l=1,\ldots ,n\right\}$,

(108) $$A_{pq}=\frac{1}{n}\sum_{l=1}^{n}\lambda_{l}e^{2\pi i(q-p)(l-1)/n}.$$

A submatrix ${\left[A\right]}_{\left\{i_{k}|k=1,\ldots ,m\right\}}$ of the matrix *A* is a (n−m)×(n−m) matrix obtained from *A* by deletion of *m* rows and *m* columns which intersect at the diagonal entries $A_{i_{k}i_{k}}$ specified by *m* integers $i_{k}\in \left[1,n\right]$. The Schur matrix $S_{pj}=e^{2\pi ipj/n}$ is employed in the fast Fourier transform and sometimes is called the Fourier matrix. A n×n degenerate Schur/Fourier matrix $S_{\nu }$ is the Schur matrix $\left(S_{pj}\right)$ each, *j*-th column of which is replicated $\nu_{j}$ times,

(109) $${\left(S_{\nu }\right)}_{pq}=e^{\frac{2\pi ipf_{\nu }\left(q\right)}{n}},\;f_{\nu }\left(q\right)=1+\sum_{t=1}^{n-1}\theta \left(q-1-\sum_{j=1}^{t}\nu_{j}\right).$$

(The multiplicities, $\nu_{j}\geq 0$, are integers; j=1,…,n.) It is specified by a *n*-tuple $\nu =\left(\nu_{1},\ldots ,\nu_{n}\right),\sum_{j=1}^{n}\nu_{j}=n$; θ(x) is a step-function. All J+1 different columns j=j(i) of $S_{\nu }$ are enumerated in the increasing order j(0)<j(1)<…<j(J) by an index i=0,1,…,J;J>0. $S_{\nu \{i_{k}\}}$ is the (n−m)×(n−m) matrix obtained from $S_{\nu }$ by deleting *m* rows with indexes $p=i_{k}\;\left(k=1,\ldots ,m\right)$ and truncating the column-index range to q=1,…,n−m.

The Laplace integral representation of the permanent of the degenerate Schur/Fourier matrix in Equation (107) involves a symmetric homogeneous polynomial in *Q* variables $q_{k}$ of degree $n-\nu_{j(0)}$ with *Q* parameters $m_{k}$,

(110) $$f_{Q}=\frac{{\left(-1\right)}^{Q}}{Q!}\prod_{i=1}^{J}{\left[\sum_{k=1}^{Q}q_{k}x_{k}^{j(i)-j(0)}\right]}^{\nu_{j(i)}},\;x_{k}=e^{\frac{2\pi im_{k}}{n}},$$

—actually, its reduced counterpart ${\tilde{f}}_{Q}\left(\left\{q_{k},m_{k}\right\}\right)$ which is built from the polynomial $f_{Q}\left(\left\{q_{k},m_{k}\right\}\right)$ by keeping only those monomials which simultaneously depend on all *Q* variables $q_{k}$; $\eta ={\left(-1\right)}^{\left(n+1\right)j\left(0\right)+n-\nu_{j(0)}}\left(\nu_{j(0)}!\right)$. The polynomial (110) can be written in terms of a special function,

(111) $$f_{Q}\left(\left\{q_{k},m_{k}\right\}\right)=\frac{{\left(-1\right)}^{Q}q_{Q}^{n}}{Q!{\left(\sum_{k=1}^{Q}q_{k}\right)}^{\nu_{j(0)}}}{\left(\{-\frac{q_{k}}{q_{Q}};\frac{x_{k}}{x_{Q}}\}\right)}_{n}^{\left\{\nu_{j}\right\}},$$

that is, a multivariate ν-generalized q-Pochhammer symbol

(112) $${\left(\left\{a_{k};q_{k}\right\}\right)}_{n}^{\left\{\nu_{j}\right\}}=\prod_{j}{\left(1-\sum_{k=1}^{Q-1}a_{k}q_{k}^{j-j(0)}\right)}^{\nu_{j}}.$$

A normal q-Pochhammer symbol of *q*-analysis [60,61] is

(113) $${\left(a;q\right)}_{n}=\prod_{j=1}^{n}\left(1-aq^{j-1}\right).$$

The number *Q* of variables $q_{k}$ in the polynomials ${\tilde{f}}_{Q}\left(\left\{q_{k},m_{k}\right\}\right)$ contributing to $\mathrm{per}S_{\nu }$ in Equation (107) depends on the *n*-tuple ν of the degenerate Schur/Fourier matrix $S_{\nu }$ but, in any case, is bounded from above by an inequality $Q\leq Q_{\max}\leq \sum_{j=1}^{n}j\nu_{j}/n-j\left(0\right),\left(n-\nu_{j(0)}\right)/2$. Equation (107) proves that only very few degenerate Schur/Fourier matrices have the nonzero permanent $\mathrm{per}S_{\nu }\neq 0$, namely, the matrices $S_{\nu }$ satisfying the following Diophantine equation $\sum_{j=1}^{n}j\nu_{j}=0\;\;\mathrm{mod}\;n$.

The number-theoretic functions discussed above in conjunction with the permanent $\mathrm{per}S_{\nu }$ are related to the prime numbers, namely, the numbers coprime to n/d, where *d* is a divisor of the matrix size *n*. The Euler’s totient function φ(k) counts the number of positive integers *j* up to a given integer *k* that are relatively prime: j≤k and the greatest common divisor (j,k)=1 is unity. The M$\ddot{o}$bius function $\mu \left(k\right)=\sum_{j=1;(j,k)=1}^{k}e^{2\pi ij/k}$ is the sum of the primitive k-th roots of unity and takes on the three values μ(k)=−1,0,1. Similarly, the Ramanujan’s sum $c_{k}\left(m\right)=\sum_{j=1;(j,k)=1}^{k}e^{2\pi imj/k}$ is the sum of the m-th powers of the primitive k-th roots of unity. The Euler’s totient function and the Ramanujan’s sum can be calculated via the M$\ddot{o}$bius function as the following sums

(114) $$\varphi \left(k\right)=\sum_{d|k}\mu \left(\frac{k}{d}\right)d,\;c_{k}\left(m\right)=\sum_{d|(k,m)}\mu \left(\frac{k}{d}\right)d,$$

and are related to the greatest common divisor (j,n) via the discrete Fourier transform:

(115) $$\varphi \left(k\right)=\sum_{j=1}^{k}\left(j,k\right)e^{\frac{2\pi ij}{k}},\;\left(j,n\right)=\sum_{m=1}^{n}e^{\frac{2\pi imj}{n}}\sum_{k|n}\frac{c_{k}\left(m\right)}{k}.$$

These facts explain why a close relation between the number-theoretic functions and the permanent [1,2,3,4] of the degenerate Schur/Fourier matrices is so natural. It is known that a computational complexity of the Euler’s totient function is that of a factoring of an integer into a product of prime numbers. It is believed that the latter problem belongs to a NP-intermediate class, that is, it is much harder than any polynomial, P-class problem, but it is not one of the hardest, NP-complete problems. The factoring of large numbers constitutes a basis for a famous RSA public-key cryptosystem and as such had been studied in detail for more than four decades. There is no efficient, polynomial-time algorithm for solving this problem, although the running time of the best known algorithm, the general number field sieve (GNFS) algorithm, for factoring a *b*-bit number is sub-exponential ∼$O(\exp\left\{4{\left[b{\left(\log\;b\right)}^{2}/9\right]}^{1/3}\right\})$.

**Figure 14:**
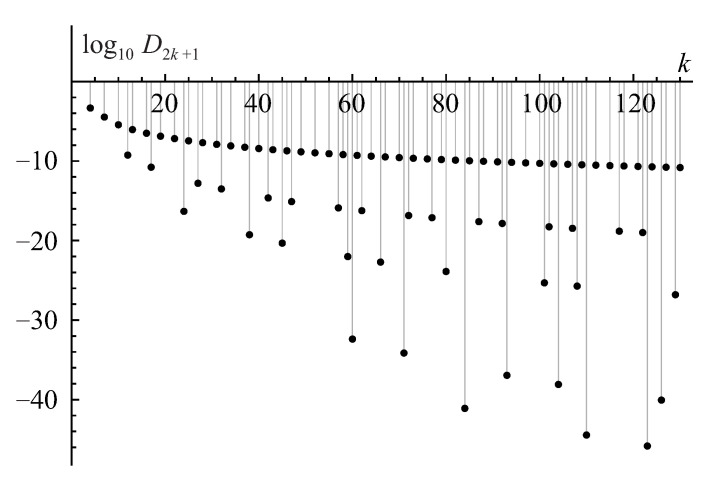
The function $D_{n}$ in Equation (116) in a logarithmic scale, $\log_{10}D_{2k+1}$, for the non-prime odd values of the integer variable n=2k+1 in the interval k=4,…,130; $D_{n}=0$ for prime *n*.

Apparently, the computational complexity of the number-theoretic functions contributes to the permanent’s computational complexity. At the same time, the latter includes also other factors. Even for the quite special Schur/Fourier matrix *S*, computing the permanent [62], $\mathrm{per}S=\sum_{d|n}u_{n}\left(d\right)\mu \left(\frac{n}{d}\right)$, requires computing a cardinality $u_{k}\left(d\right)$ of a subset of permutations $\left\{\pi \in S_{n}|\sum_{l=1}^{n}l\pi \left(l\right)=d\;\mathrm{mod}\;n\right\}$, along with the M$\ddot{o}$bius function μ(n/d). Moreover, even a contribution of the first two orders (Q=1,2) in Equation (107), $\mathrm{per}S=n\left(n-2\right)!\left(H_{n-2}-2+D_{n}\right)+\left\{\mathrm{terms}\;\mathrm{of}\;\mathrm{order}\;Q\geq 3\right\}$, includes the nontrivial number-theoretic functions: A harmonic number $H_{m}=\sum_{k=1}^{m}1/k$ and a new function [59]

(116) $$D_{n}=\frac{1}{2(n-1)}\sum_{1<m<n;(n,m)\neq 1}\sum_{k=1}^{(n,m)-1}\frac{C_{\left(n,m\right)}^{k}}{C_{n-2}^{kn/(n,m)-1}},$$

involving the greatest common divisor (n,m) via the binomial coefficients $C_{p}^{q}=p!/\left(q!\left(p-q\right)!\right)$ and shown in Figure 14 in the logarithmic scale. (Note a remarkable series of branches emerging at increasing values of the argument *n*.)

For the degenerate Schur/Fourier matrices $S_{\nu }$ specified by the *n*-tuples $\left\{\nu_{1},\ldots ,\nu_{n}\right\}$ or the circulant matrices specified by the eigenvalues $\left\{\lambda_{1},\ldots ,\lambda_{n}\right\}$ (see Equation (106)), the permanent’s computational complexity greatly increases due to a necessity to compute $\mathrm{per}S_{\nu }$ for a very large number $r\left(n\right)=\sum_{d|n}\varphi \left(\frac{n}{d}\right)\frac{(2d-1)!}{d!(d-1)!n}$ of the different *n*-tuples ν generating the nonzero permanents [63] $\mathrm{per}S_{\nu }$, even though this number is much less than the total number of all degenerate Schur/Fourier matrices $T_{n}=\left(n+1\right)C_{n}/2$, where $C_{n}=\left(2n\right)!/\left[\left(n+1\right)!n!\right]$ is the Catalan number. This additional complexity is encrypted into the polynomial (110) of the permanent’s integral representation (107) via the other special function—the multivariate ν-generalized q-Pochhammer symbol (112).

An overall complexity of the degenerate Schur/Fourier matrix permanent can be understood as a combinatorial, number-theoretic complexity of the multiset partitions $\nu^{Q}=\left\{\nu^{\left(k\right)}|k=1,\ldots ,Q\right\}$ of the *n*-tuple $\nu =\{\nu_{1},\ldots ,\nu_{n}\}$ which constitute the matrix’s discrete representation [59]

(117) $$\mathrm{per}S_{\nu }=\frac{\eta \prod_{j=1}^{n}\nu_{j}!}{\nu_{j(0)}!}\sum_{Q=1}^{Q_{\max}}\sum_{\nu^{Q}}\frac{{\left(-n\right)}^{Q}}{\prod_{l=1}^{L}\left(d_{l}!\right)}\prod_{k=1}^{Q}\frac{(M_{k}-1)!}{\prod_{i=1}^{J}\left(\nu_{j(i)}^{\left(k\right)}!\right)}.$$

Here *Q* submultisets $\nu^{\left(k\right)}=\left\{\nu_{j(1)}^{\left(k\right)},\ldots ,\nu_{j(J)}^{\left(k\right)}\right\}$ partition the multiset $\nu^{\prime }=\left\{\nu_{j(1)},\ldots ,\nu_{j(J)}\right\}$ of all, except $\nu_{j(0)}$, nonzero multiplicities $\nu_{j(i)}$ of a given *n*-tuple $\nu =\{\nu_{1},\ldots ,\nu_{n}\}$ into *Q* summands, that is, $\nu_{j(i)}=\sum_{k=1}^{Q}\nu_{j(i)}^{\left(k\right)}$. They are naturally ordered, $\nu^{\left(k\right)}⪰\nu^{\left(k+1\right)}$, in accord with the decreasing order $\nu_{j(i)}^{\left(k\right)}\geq \nu_{j(i)}^{\left(k+1\right)}$ of their components. The lengths $d_{l}$(l=1,…,L) of the intervals of equal consecutive submultisets $\nu^{\left(k_{l}\right)}=\nu^{\left(k_{l}+1\right)}=\ldots =\nu^{\left(k_{l}+d_{l}-1\right)}$ are called the degeneracy factors of the multiset partition $\nu^{Q}$. Each submultiset $\nu^{\left(k\right)}$ covers a total number $M_{k}=\sum_{i=1}^{J}\nu_{j(i)}^{\left(k\right)}\leq \sum_{i=1}^{J}\nu_{j(i)}=n-\nu_{j(0)}$ of columns in the matrix $S_{\nu }$. The sum in Equation (117) runs over those multiset partitions $\nu^{Q}$ for which all submultiset partitions $\nu^{\left(k\right)}$ have a span $\sigma \left(\nu^{\left(k\right)}\right)=\sum_{i=1}^{J}\left(j\left(i\right)-j\left(0\right)\right)\nu_{j(i)}^{\left(k\right)}$ divisible by *n*, that is, $\sigma (\nu^{\left(k\right)})=0\;\mathrm{mod}\;n\;\forall \;k=1,\ldots ,Q$.

Thus, the permanent, $\mathrm{per}S_{\nu }$, of the degenerate Schur/Fourier matrix, both in the form of the Laplace integral representation in Equation (107) and in the equivalent form of the discrete representation in Equation (117), is the sum over the multiset partitions which constitute a major focus in the number theory and combinatorics.

In addition to the aforementioned functions related to $\mathrm{per}S_{\nu }$, the other combinatorial and number-theoretical functions enter the scene when one calculates the permanent of the circulant matrix via the power expansion over the matrix eigenvalues in Equation (106). This happens because the latter includes the sum over the *n*-tuples $\nu =\{\nu_{1},\ldots ,\nu_{n}\}$ specifying the degenerate Schur/Fourier matrices, in addition to the aforementioned sum over the multiset partitions. In particular, the number of terms with a given value of the exponent $\nu_{1}$ in $\lambda_{1}^{\nu_{1}}$ is equal to the well-known rencontres (encounter) numbers [53]

(118) $$D_{n,\nu_{1}}=\left(!\left(n-\nu_{1}\right)\right)C_{n}^{\nu_{1}}=\frac{n!}{\nu_{1}!}\sum_{k=0}^{n-\nu_{1}}\frac{{\left(-1\right)}^{k}}{k!},\;!n\equiv \lceil \frac{n!}{e}\rfloor ;$$

⌈x⌋ means rounding *x* up for even *n* and down for odd *n*.

## 7. Asymptotics of the Permanent and the Szegő Limit Theorems

The result (2) points to a fundamental open problem of finding the permanent’s asymptotics for the case of the large-size circulant matrix and its analogy with the Szegő limit theorems on the Toeplitz determinant employed in the Onsager’s exact solution of the 2d Ising model [47,48,49,50,51,52].

A starting point for finding this asymptotics could be the expansion (106) of perA in powers of the A’s eigenvalues.

An interesting example of an asymptotic reduction of the permanent of a doubly stochastic n×n matrix *A*, with a moderate variation of its entries, to the determinant,

(119) $$\mathrm{per}\left(nA\right)∼n!/\sqrt{\det(I+J-A^{\prime }A)}\;\mathrm{at}\;\;n\to \infty ,$$

was found in Reference [64]; $I_{pq}=\delta_{p,q}$, $J_{pq}=1/n$, $A^{\prime }$ stands for the transpose of *A*. It works quite well, for example, for the nonnegative circulant matrices with a power-law, $a_{q}∼q^{-\xi }$, or similar moderate variation of entries. Other ways to estimate the permanent have been recently reviewed in Reference [4].

For the symmetric circulant matrix *A*, the matrix under the determinant in Equation (119) is circulant, that is Toeplitz. Below we show that the permanent’s asymptotics (119) is directly related to the Toeplitz-determinant asymptotics.

### 7.1. The Circulant Determinant vs. the Toeplitz Determinant

Finding the permanent’s asymptotics for the large-size circulant matrix remains an open problem who’s solution would be the key to a practical application of the permanents in the theory of critical phenomena and quantum many-body processes as well as related studies of the nature’s complexities. An analogous problem of finding the asymptotics of the Toeplitz determinant was the key to the Onsager’s solution of the 2d Ising model [47,48,49,50,51,52]. The latter problem had been solved by the first and second Szegő limit theorems on the asymptotics of the Toeplitz determinant. Here we elaborate on this analogy in view of the McCullagh permanent’s asymptotics [64] in Equation (119) applied to the symmetric circulant matrix A. In this case the problem is reduced to finding the determinant’s asymptotics of the circulant and, hence, Toeplitz matrix $I+J-A^{\prime }A$ where $A^{\prime }$ is the transpose of A. This fact sets a direct relation between the permanent’s asymptotics and the Szegő limit theorems.

This relation is based on an important fact that the determinant of the n×n circulant matrix $C_{n}=\left(c_{q-p\;\mathrm{mod}n}\right)$ can be explicitly computed as a product of its eigenvalues

(120) $$\lambda_{l}=\sum_{k=0}^{n-1}c_{k}e^{-2\pi ikl/n},\;l=0,1,\ldots ,n-1,$$

given by a discrete Fourier transform of the first row of the circulant matrix, ${\left(C_{n}\right)}_{1q}=c_{q-1},q=1,\ldots ,n$. Assuming that all of the eigenvalues are not zero, one has

(121) $$\det\;C_{n}=\exp\left[\sum_{l=0}^{n-1}\log\lambda^{\left(n\right)}\left(e^{-2\pi il/n}\right)\right];\;\lambda^{\left(n\right)}\left(z\right)=\sum_{k=0}^{n-1}c_{k}z^{k}.$$

Here an associate polynomial $\lambda^{\left(n\right)}\left(z\right)$ of the circulant matrix $C_{n}$ is taken on the unit circle in the complex plane, $z=e^{-2\pi ix}$, at the discrete, homogeneously distributed values of a polar angle 2πx corresponding to the values $x_{l}=l/n,l=0,1,\ldots ,n-1$ of a real variable x∈[0,1].

The exact Equation (121) is a discrete analog of the first Szegő limit theorem [51]. The latter gives a leading term of the determinant’s asymptotics for the Toeplitz n×n matrix $T_{n}=\left(t_{q-p}\right)$ at n→∞ via a mean value of the logarithm of its associate polynomial, symbol t(z), on the unit circle:

(122) $$\lim_{n\to \infty }{\left(\det\;T_{n}\right)}^{\frac{1}{n}}=e^{\int_{0}^{1}\log t\left(e^{-2\pi ix}\right)dx};\;t\left(z\right)=\sum_{k=-\infty }^{\infty }t_{k}z^{k}.$$

This leading asymptotic behavior corresponds to the homogeneous distribution of the eigenvalues on the unit circle.

The next-to-leading term in the Toeplitz-determinant asymptotics, namely, the independent-on-*n* pre-factor in front of the leading term (that, according to Equation (122), has an exponent growing linearly with *n*) is given by the second (strong) Szegő limit theorem as follows

(123) $$\lim_{n\to \infty }\frac{\det\;T_{n}}{e^{n\int_{0}^{1}\log t\left(e^{-2\pi ix}\right)dx}}=\exp\left[\sum_{k=1}^{\infty }k{\left(\log t\right)}_{k}{\left(\log t\right)}_{-k}\right],$$

where ${\left(\log t\right)}_{k}=\int_{0}^{1}e^{-2\pi ikx}\log\left[t\left(e^{2\pi ix}\right)\right]dx$ is the *k*-th Fourier coefficient of the logarithm of the symbol t(z) associated with a limit Toeplitz matrix $T=\lim_{n\to \infty }T_{n}$.

On the one hand, an appearance of the nontrivial factor (123) due to the strong Szegő theorem manifests a more complex structure of the Toeplitz matrices in the general case compared to the structure of a special subset of the Toeplitz matrices - the circulant matrices. Essentially, a product of two circulant matrices is always the circulant matrix, while the Toeplitz matrices in the general case lack this property of a multiplicativity, that is, do not form a multiplicative group [65]. On the other hand, among the Toeplitz matrices there is a wide subset of matrices for which the symbol t(z) is well defined, for instance, when a well-known sufficient condition for a convergence of its series in Equation (122), $\sum_{k}\left|t_{k}\right|<\infty$, is satisfied due to a relatively fast decrease of the entries $t_{\pm k}$ at k→±∞. The Szegő limit theorems are directly applicable only to this subset of the Toeplitz matrices.

For the circulant matrices $C_{n}$, a definition of the associate Toeplitz symbol employed in Equation (122) is not appropriate since a series $t_{n}^{\left(C\right)}\left(z\right)=c_{0}+\sum_{k=1}^{n-1}\left(c_{k}z^{k}+c_{n-k}z^{-k}\right)$ is not convergent at n→∞. The definition of the symbol,

(124) $$\lambda \left(z\right)=\sum_{k=0}^{\infty }c_{k}z^{k},$$

based on the associate polynomial $\lambda^{\left(n\right)}\left(z\right)$ introduced in Equation (121) could be used, but only if the sequence $c_{k}$ is decreasing so that, for instance, the convergence condition $\sum_{k}\left|c_{k}\right|<\infty$ is satisfied. However, it would exclude from consideration the basic case of the periodic boundary conditions for the phase transition problem when the first row of the circulant matrix, that is, the correlation function, has the same values at the symmetrically located positions, $|c_{k}\left|=\right|c_{n-k}|$. In the latter case, let us define a circulant-matrix symbol $\bar{\lambda }\left(z\right)$ via a modified associate polynomial which monomials $z^{k}$ acquire a shift −n of the exponent *k* if it is greater than an integer part of n/2,

(125) $$\bar{\lambda }\left(z\right)=\lim_{n\to \infty }{\bar{\lambda }}^{\left(n\right)},\;{\bar{\lambda }}^{\left(n\right)}\left(z\right)=\sum_{k=0}^{\left[n/2\right]}c_{k}z^{k}+\sum_{k=[n/2]+1}^{n-1}c_{k}z^{k-n}.$$

The latter preserves the circulant determinant (121):

(126) $$\det\;C_{n}=\exp\left[\sum_{l=0}^{n-1}\ln{\bar{\lambda }}^{\left(n\right)}\left(e^{-2\pi il/n}\right)\right].$$

The most interesting, nontrivial situation of passing the critical region of a phase transition corresponds to the case when the related circulant matrix entering the result in Equation (2) and discussed in Section 2 above ceases to have a convergent symbol, neither λ(z) nor $\bar{\lambda }\left(z\right)$. This occurs in the critical region of parameters when the correlation function representing the first row of this circulant matrix experiences a transition from an abrupt exponential decay to spreading over an entire macroscopic volume of the system. In this case computing the asymptotics of the circulant determinant amounts to finding the asymptotics of the series in the exponent of Equation (121) or Equation (126). Note that even then this series could be represented sometimes as an integral from a smooth function over the unit interval x∈[0,1]. The latter function may be different from the logarithm of the polynomial $\lambda^{\left(n\right)}\left(z\right)$ or ${\bar{\lambda }}^{\left(n\right)}\left(z\right)$ associated with the circulant matrix as per Equation (121) or Equation (126) since this polynomial is strongly oscillating, ill-defined at n→∞. An example is given below.

The aforementioned nontrivial, complimentary relation between the asymptotics of the circulant determinant in terms of the explicit exact formula in Equation (121) or Equation (126) and the asymptotics of the Toeplitz determinant in terms of the Szegő limit theorems could be clarified by considering a typical case of the circulant matrix being represented as a sum of two Toeplitz matrices, $C_{n}=T_{n}+{\bar{T}}_{n}$, who’s entries $t_{\pm k}$ or ${\bar{t}}_{\pm k}$ generally decrease in value with increasing *k* or n−k from 1 to n−1, respectively. Then, the determinant of the circulant matrix $C_{n}$ differs from the determinant of the Toeplitz matrix $T_{n}$ by a factor:

(127) $$\det C_{n}=\det T_{n}\;\det\left(1+{\bar{T}}_{n}T_{n}^{-1}\right).$$

Let’s consider a symmetric circulant matrix with the entries $c_{k}=c_{n-k}$ decreasing in absolute value when *k* runs from 1 to [n/2]. Let the Toeplitz matrix $T_{n}$ contains the central (1+2[n/2])-band of the circulant matrix $C_{n}$ and zero entries in the complimentary to this band upper and lower triangular parts, that is, $t_{k}=c_{k}$ for k=0,1,…,[n/2], $t_{k}=c_{n+k}$ for k=−1,…,−[n/2], and $t_{k}=0$ for |k|>[n/2]. Suppose $\sum_{k}\left|t_{k}\right|<\infty$, so that the Toeplitz symbol t(z) in Equation (122) exists and the Szegő limit theorems in Equations (122) and (123) apply. Then, since the Toeplitz-matrix symbol t(z) coincides with the circulant-matrix symbol $\bar{\lambda }\left(z\right)$ as per Equations (125) and (126), the circulant determinant in the left hand side of Equation (127) coincides with the leading term of the Toeplits-determinant asymptotics given by the first Szegő theorem (122). Obviously, the second (strong) Szegő theorem (123) gives the pre-factor $1/\det(1+{\bar{T}}_{n}T_{n}^{-1})$ that accounts for a fact that computing the Toeplitz determinant cannot be reduced to computing a mean value of the logarithm of the symbol over the homogeneous distribution of eigenvalues on the unit circle and, hence, is more complex than computing the circulant determinant. Only in the case when a contribution due to the complimentary Toeplitz matrix ${\bar{T}}_{n}$ becomes negligible, the strong Szegő theorem (123) is reduced to just a trivial, unity factor and, hence, the Toeplitz determinant is reduced to a value given by the first Szegő theorem and coinciding with the exact result in Equation (121) or (126) for the circulant determinant.

### 7.2. McCullagh Asymptotics of the Permanent and Two Opposite Limits for the Circulant Determinant

Now we are ready to unfold the permanent’s asymptotics (119) at large *n* for the doubly stochastic matrix $A_{n}=C_{n}/\lambda_{1}$ with a moderate variation of its entries,

(128) $$\frac{n^{n}\mathrm{per}A_{n}}{n!}∼\frac{1}{\sqrt{\det(I+J-A_{n}^{\prime }A_{n})}}\;.$$

The eigenvalues of the matrix $A_{n}=C_{n}/\lambda_{1}$ differ from that of the matrix $C_{n}$, Equation (120), only by the scaling factor $\sum_{k=0}^{n-1}c_{k}=\lambda_{1}$. Each eigenvalue of the matrix $I+J-A_{n}^{\prime }A_{n}$ entering the McCullagh asymptotics equals to the sum of the eigenvalues of matrices I,J, and $-A_{n}^{\prime }A_{n}$ since all of these matrices are circulant in the case of the symmetric circulant matrix *A* considered here and, hence, can be diagonalized by the same discrete Fourier transformation. Thus, the determinant of the matrix $I+J-A_{n}^{\prime }A_{n}$ is given by the product of its eigenvalues:

(129) $$\det\left(I+J-A_{n}^{\prime }A_{n}\right)=\prod_{k=2}^{n}\left(1-\frac{\lambda_{k}^{2}}{\lambda_{1}^{2}}\;\right),\;A_{n}=\frac{C_{n}}{\lambda_{1}}.$$

Here we implemented the fact that only the first eigenvalue of the matrix *J* is not zero, namely, it equals unity. As a result, calculating the product in Equation (129) via the exact eigenvalues in Equation (120), we get the explicit asymptotics of the permanent by means of Equation (128). Since the entries $c_{k}$ and the eigenvalues $\lambda_{l}$ of a sequence of the circulant matrices $\left\{C_{n}\right\}$ constitute two counterparts of the discrete Fourier transform as per Equation (120), there are two opposite limiting cases. Namely, either

(i) the matrix entries given by the function $c_{k}$ of the integer variable *k* enumerating matrix columns are well localized and do not spread over an entire range of the column index *k* with increasing matrix size *n*, for example, when the conversion condition $\sum_{k}\left|c_{k}\right|<\infty$ is satisfied, or

(ii) the eigenvalues $\lambda_{l}$ are similarly well localized and do not spread over an entire range of the eigenvalue index *l* with increasing matrix size *n*.

In the first case, the limiting distribution of the eigenvalues $\lambda_{l}$ could be described by a smooth, well-defined function, say, $\lambda (e^{2\pi ix})$ or $\bar{\lambda }\left(e^{2\pi ix}\right)$, of the variable x=l/n∈[0,1]. In the second case, the limiting distribution of the entries $c_{k}$ could be described by a smooth, well-defined function of the variable x=k/n∈[0,1].

This alternative is a manifestation of the uncertainty principle: The more concentrated a function is, the more spread out its Fourier transform must be. In the application to the critical phenomena in a spontaneous symmetry breaking, a transition from the first case to the second case corresponds to the transition from a disordered phase with a strongly localized correlation function to an ordered phase with the correlation function spread out over an entire, macroscopic dimension of a many-body system. The complexity of the critical phenomena revealed by the matrix permanent’s complexity arises due to a simultaneous absence of such smooth, well-defined functions in the both dual domains of the matrix entries and the eigenvalues that takes place in the central part of the critical region of phase transitions.

Let us illustrate a related transition in the behavior of the permanent’s asymptotics by considering a sequence of the symmetric circulant n×n matrices $\left\{C_{n}\right\}$ who’s first rows $c_{k}$ are given by smooth functions $c^{\left(n\right)}\left(x\right)$ of the continuous variable x∈[0,1] at the discrete points x=k/n. In accordance with the Poisson summation formula for a discrete-time Fourier transform (DTFT), the eigenvalues are given by Equation (120) as a periodic summation of Fourier coefficients ${\tilde{c}}^{\left(n\right)}\left(j\right)$ of the function $c^{\left(n\right)}\left(x\right)$ as follows

(130) $$\lambda_{l}=n\sum_{j=-\infty }^{\infty }{\tilde{c}}^{\left(n\right)}\left(l+nj\right),\;{\tilde{c}}^{\left(n\right)}\left(l\right)=\int_{0}^{1}c^{\left(n\right)}\left(x\right)e^{-2\pi ilx}dx.$$

The determinant of the matrix $C_{n}$ equals their product:

(131) $$\det C_{n}=n^{n}\prod_{l=0}^{n-1}\sum_{j=-\infty }^{+\infty }{\tilde{c}}^{\left(n\right)}\left(l+nj\right).$$

In a class of the functions $c^{\left(n\right)}\left(x\right)=h\left(\beta_{n}x\right)$ which are given by one and the same smooth function h(x),x∈[0,∞), and depend on the matrix size *n* only via a scaling factor $\beta_{n}$, the first of the two opposite limiting cases discussed above can be illustrated by the following typical case of the scaling $\beta_{n}=n$. If the function h(x) is decreasing fast enough at large values of |x|, then, in the limit of large *n*, the matrix $C_{n}$ becomes essentially a relatively narrow band matrix and the entries $c_{k}\equiv c^{\left(n\right)}\left(x=k/n\right)=h\left(k\right)$ constitute the Fourier coefficients of the well-defined symbol $\bar{\lambda }\left(z\right)$, Equation (125). So, the associated polynomials ${\bar{\lambda }}^{\left(n\right)}\left(z\right)$ of the matrices $C_{n}$ could be considered as projections of the symbol $\bar{\lambda }\left(z\right)$ onto a subspace of polynomials spanned by the finite-power monomials $z^{k},\;k\in \left[\left[\frac{n}{2}\right]+1-n,\left[\frac{n}{2}\right]\right]$. If the symbol $\bar{\lambda }\left(z\right)$ is a smooth positive function, so that the projections ${\bar{\lambda }}^{\left(n\right)}\left(z\right)$ tends to $\bar{\lambda }\left(z\right)$ with increasing *n*, then the eigenvalues $\lambda_{l}$ tend to $\bar{\lambda }\left(e^{2\pi il/n}\right)$ in a dense and homogeneous set of points on the unit circle. In this case, the leading contribution to the determinant in Equation (130) is provided solely by the Fourier coefficients within the central *n*-period of harmonics, $l+nj\in [-n+1+\left[\frac{n}{2}\right],\left[\frac{n}{2}\right]]$, and the asymptotics of the determinant is given by the integral of the logarithm of the symbol over the unit circle:

(132) $$\lim_{n\to \infty }\det C_{n}=\exp\;\left[n\int_{0}^{1}\ln\bar{\lambda }\left(e^{2\pi ix}\right)dx\right].$$

This formula for the determinant of circulant matrices has the same form as the first Szegő limit theorem in Equation (122). Note, however, that the analogous asymptotics of the Toeplitz determinant includes an additional nontrivial pre-exponential factor given by the strong Szegő theorem, Equation (123). The reason for this difference was discussed above, after Equation (127), and stems from the fact that the circulant and Toeplitz matrices are close to each other only in terms of the weak or Hilbert-Schmidt norm, but differ significantly in terms of the strong norm [51]. Hence, their determinants have different limiting values.

The second of the two opposite limiting cases discussed above can be illustrated by the case of the unity scaling factor $\beta_{n}=1$. Now, with increasing *n*, the entries $c_{k}\equiv c^{\left(n\right)}\left(x=k/n\right)=h\left(k/n\right)$ of the circulant matrices $C_{n}$ tend to the values of the smooth function h(x) on a dense homogeneous set of points {k/n} within the unit interval x∈[0,1]. In virtue of the uncertainty principle, it implies that the eigenvalues $\lambda_{l}$ determined by the entries’ Fourier transform as per Equation (130) appear to be strongly localised in the dual, frequency domain. In the case of the symmetric doubly-stochastic matrix $A_{n}=C_{n}/\lambda_{1}$ with the entries $c_{k}$ decreasing from the side columns k=0 and k=n towards the central column k=[n/2], which corresponds to the problem of phase transition in a system with the periodic boundary conditions and is relevant to the McCullagh permanent’s asymptotics, the generating function h(x) is real-valued and positive. So, at n→∞, all of the significant eigenvalues are concentrated only near the boundaries l=1 and l=n, that is, at l≪n and n−l≪n, as the symmetric pairs $\lambda_{l}=\lambda_{n-l}$. In the leading order, their asymptotics is given by just one relevant term ${\tilde{c}}^{\left(n\right)}\left(l\right)={\tilde{c}}^{\left(n\right)}\left(n-l\right)$ in the periodic summation in Equation (130). The rest of the eigenvalues, spanning the entire central part of the range of Fourier harmonic numbers *l* between these boundary layers, tend to zero. As a result, the McCullagh permanent’s asymptotics in Equations (128) and (129) acquires the following simple form

(133) $$\lim_{n\to \infty }\frac{n^{n}\;\mathrm{per}A_{n}}{n!}=\prod_{l=2}^{\infty }\frac{1}{1-{\left[{\tilde{c}}^{\left(n\right)}\left(l\right)/{\tilde{c}}^{\left(n\right)}\left(1\right)\right]}^{2}}\;.$$

### 7.3. An Example of the Permanent’s Asymptotics: Circulant Matrix with Exponentially Varying Entries

As an example, let us consider the circulant matrices $C_{n}$ who’s first row,

(134) $$c_{k}=\cosh\left(\alpha -2\alpha k/n\right),\;k=0,1,\ldots ,n-1,$$

corresponds to an exponential decay of correlations described by a nonnegative parameter α and the periodic boundary conditions. In this case the discrete Fourier transform (120) yields the eigenvalues explicitly:

(135) $$\lambda_{k}=\frac{\sinh(\alpha )\sinh(2\alpha /n)}{\cosh(2\alpha /n)-\cos(2\pi (k-1)/n)},\;k=1,2,\ldots ,n.$$

A straightforward calculation of the product in Equation (129) yields the explicit asymptotics of the permanent via Equation (128) for the large finite values of *n*. Here we present it only for n≫1+α when the determinant gets to the limit

(136) $$\lim_{n\to \infty }\det\left(I+J-A_{n}^{\prime }A_{n}\right)=\frac{\alpha^{2}\sinh^{2}\left(\alpha \sqrt{2}\right)}{2\;\sinh^{4}\left(\alpha \right)}$$

and the leading term of the permanent’s asymptotics is

(137) $$\lim_{n\to \infty }\frac{n^{n}\mathrm{per}A_{n}}{n!}=\frac{\sqrt{2}\;\sinh^{2}\left(\alpha \right)}{a\;\sinh(\alpha \sqrt{2})}.$$

The latter result follows from Equation (133), that is, calculation of the product in Equation (129) as ${\left(\prod_{k=2}^{r}\right)}^{2}$ for r=[n/2]→∞, by means of the integration over a parameter p∈[0,1],

(138) $$\prod_{k=1}^{r}\left(1+\frac{u}{v+\pi^{2}k^{2}}\right)=\exp\int_{0}^{1}\sum_{k=1}^{r}\frac{u\;dp}{pu+v+\pi^{2}k^{2}},$$

via a sum given by a digamma function $\psi \left(z\right)=\frac{d[\ln\Gamma (z)]}{dz}$,

(139) $$\sum_{k=1}^{r}\frac{1}{x^{2}+k^{2}}=\frac{\pi x\coth(\pi x)-1}{2x^{2}}-\frac{\psi (r+ix)-\psi (r-ix)}{2ix}.$$

**Figure 15:**
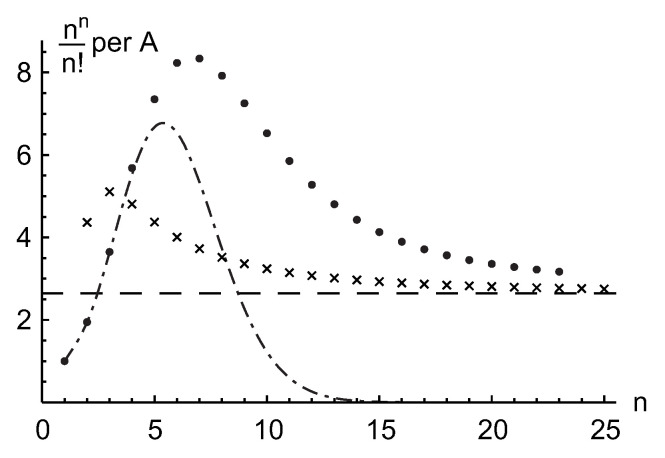
The scaled permanent, $n^{n}\mathrm{per}A_{n}/n!$, of the doubly stochastic circulant n×n matrix $A_{n}=C_{n}/\lambda_{1}$ specified in Equation (134) as a function of the matrix size: the dots—an exact numerical calculation, the crosses—the McCullagh asymptotics in Equation (128), the dashed-dotted curve—the diagonal (random phase) approximation in Equation (140), the dashed line—the leading asymptotics in Equation (137); α=2.5.

Dependence of the permanent on the matrix size *n* (the exact values calculated numerically and shown by dots) and its McCullagh asymptotics (128) (shown by crosses) are illustrated in Figure 15 for the doubly stochastic circulant matrix $A_{n}=C_{n}/\lambda_{1}$ specified in Equation (134) in the case of the relatively large correlation decay parameter, α=2.5. This parameter sets a scale of the entries’ variation from the maximum value $c_{k=0}=\cosh\left(\alpha \right)\gg 1$ at the main diagonal to the minimum value at a half way to the matrix edge $c_{k=[n/2]}$ that is 1 for even *n* and about 1 for odd n>α. The fact that this scale of variation is a fixed, independent on *n* quantity makes the matrix $A_{n}$ resemble the matrix *J* at n→∞ and, hence, justifies McCullagh asymptotics which approximates the permanent quite well starting already from n∼20. At the same time, for relatively small *n*, a steep, exponential decay of matrix entries with a deviation from the main diagonal makes the matrix $A_{n}$ resemble the narrow band matrix that validates an opposite-type approximation of the permanent as the product of the diagonal entries,

(140) $$\mathrm{per}A_{n}\approx {\left(\frac{c_{0}}{\lambda_{1}}\right)}^{n}={\left[\frac{\tanh(\alpha /n)}{\tanh(\alpha )}\right]}^{n},$$

associated with a random phase approximation [33].

Thus, the example shown in Figure 15 simultaneously illustrates both opposite limiting cases (i) and (ii) of the circulant permanent behavior discussed above and a nontrivial transition between them. An unambiguous agreement of the exact numeric calculations with corresponding analytic results in Equations (140) (a dashed-dotted curve) and (137) (a dashed line) at small (n<5) and large (n>20) matrix sizes *n*, respectively, is remarkable.

### 7.4. The Permanent’s Asymptotics for the Circulant Matrices with the Power-Law Varying Entries

A similar analysis of the permanent for the circulant matrices with a power-law variation of the entries, which is characteristic for emergence of the ordered phase in the critical region within the renormalization-group approach and is significantly less steep than the exponential variation in Equation (134) discussed above, also confirms the aforementioned two opposite types of the permanent behavior. Here, for the sake of room, we skip it along with an illustrative plot of the *n*-dependence of the permanent which is similar to the one shown in Figure 15.

### 7.5. An Example of the Exact Analytic Solution for the Permanent of the Doubly Stochastic Circulant Matrix

Finally, we elaborate on one more interesting example of the permanent’s asymptotics. Let us consider the circulant n×n matrix all entries of which are equal to $c_{1}$ except the main diagonal filled with the other entry $c_{0}$,

(141) $$C_{n}=\left(c_{0}-c_{1}\right)I+c_{1}J.$$

Its first eigenvalue is $\lambda_{1}=c_{0}-c_{1}+nc_{1}$. All other eigenvalues are equal to $\lambda_{k}=c_{0}-c_{1},\;k=2,\ldots ,n$. We find its permanent analytically using combinatorics and the well-known rencontres number !k of the derangements (118), that is, the permutations of the set {1,2,…,k} without fixed points:

(142) $$\mathrm{per}C_{n}=\sum_{k=0}^{n}C_{n}^{k}\left(!k\right)c_{0}^{n-k}c_{1}^{k}=c_{1}^{n}e^{\frac{c_{0}}{c_{1}}-1}\Gamma \left(n+1,\frac{c_{0}}{c_{1}}-1\right),$$

where Γ is an upper incomplete gamma function. Introducing a relative variance of the diagonal and off-diagonal entries, $\alpha_{n}=\frac{c_{0}}{c_{1}}-1$, which plays a part similar to that of the correlation decay parameter α in Equation (134), we find the exact analytic formula for the permanent of the related doubly stochastic circulant matrix $A_{n}=C_{n}/\lambda_{1}$,

(143) $$\mathrm{per}A_{n}=\frac{e^{\alpha_{n}}\Gamma \left(n+1,\alpha_{n}\right)}{{\left(n+\alpha_{n}\right)}^{n}}\;,$$

and its McCullagh asymptotics in Equations (128) and (129),

(144) $$\mathrm{per}A_{n}∼\frac{n!}{n^{n}}{\left[1-\frac{1}{{\left(1+n/\alpha_{n}\right)}^{2}}\right]}^{(1-n)/2}\;\mathrm{at}\;n\to \infty .$$

Again, there are two opposite limiting cases. If the entries’ variance parameter $a_{n}$ is very large, then the matrix $A_{n}$ is close to the diagonal matrix and its permanent is given by the diagonal (random phase) approximation

(145) $$\mathrm{per}A_{n}\approx {\left(\frac{c_{0}}{\lambda_{1}}\right)}^{n}={\left[\frac{\alpha_{n}+1}{\alpha_{n}+n}\right]}^{n}\;.$$

In the opposite case of small values of the variance parameter $\alpha_{n}$, the matrix $A_{n}$ is of the *J* type and the McCullagh asymptotics in Equation (144) applies. A transition between these two limit regimes occurs at the moderate values of $\alpha_{n}$. It is described by the exact analytic result in Equation (143) and is similar to the one discussed in Figure 15. Importantly, the exact solution in Equation (143) allows one to analytically obtain all of the permanent’s asymptotics and limits for the matrix (141) via the known asymptotics and limits of the incomplete gamma function.

A comparative analysis of various permanent’s asymptotics will be presented elsewhere.

## 8. Conclusions

We infer that the method of a matrix permanent can be employed as a universal tool for analyzing and measuring complexity in nature. A nontrivial reduction of the critical phenomena, fractals, many-body processes in quantum systems and computing of NP- and ♯P-problems to the permanent as well as the permanent’s integral representations presented above clearly demonstrate the power and capability of the permanent-based analytic technique for the unification of the nature’s complexities.

Of course, there are other functions, different from the matrix permanent, which are also involved into various ♯P- or NP-complete problems [66]. However, in view of a number of the aforementioned results and facts, the permanent is marked by its universal and central role in describing complexity both in physics and mathematics.
